# Attitudes Towards Remote Monitoring for Falls Prevention Among Staff, Patients, Residents and Families in Hospital and Aged Care: Scoping Review

**DOI:** 10.1111/jan.70647

**Published:** 2026-05-26

**Authors:** Kelly Stephen, Dai Pu, Jennifer Weller‐Newton, Ravi Manohar, Terry P. Haines

**Affiliations:** ^1^ School of Primary and Allied Health Care, Faculty of Medicine, Nursing and Health Sciences Monash University Frankston Victoria Australia; ^2^ Eastern Health Box Hill Victoria Australia; ^3^ Faculty of Medicine and Health Sciences The University of Melbourne Carlton Victoria Australia; ^4^ School of Nursing & Midwifery, Faculty of Health University of Canberra Canberra Australian Capital Territory Australia

## Abstract

**Aim:**

To examine the reasons for and methods of using remote video monitoring to prevent falls across hospital and residential aged care, and explore how staff, patients, residents and families perceive its use and benefits.

**Design:**

Scoping Review.

**Methods:**

Following JBI methodology, eight databases were searched in July 2025 with no date restrictions. Two reviewers independently screened studies using predefined criteria, and one reviewer extracted data. Narrative and thematic syntheses described how video monitoring is implemented for falls prevention and explored stakeholders' attitudes.

**Results:**

Thirty‐five studies were included, with 77% conducted in hospitals and 86% focusing on staff perspectives, highlighting a critical underrepresentation of patients/residents and families. Perceived effectiveness was shaped by underlying motivations—falls prevention, workforce optimisation, or cost reduction. Attitudes were influenced by workload impacts, video monitoring knowledge, ethical and liability concerns. Three remote video monitoring models were identified: technician‐based, automated alerts, and nurse‐observed without alerts. Technician‐based systems were only in hospitals, with no equivalent in aged care.

**Conclusion:**

Research on remote video monitoring for falls prevention is heavily weighted towards hospitals and staff perspectives. Nurses generally viewed video monitoring as effective but still preferred in‐person observers. Although there is interest in innovative monitoring systems in aged care that balance safety with a homelike environment, empirical research is lacking. Patient, resident, and family experiences remain underrepresented and require further research.

**Impact:**

Remote video monitoring has emerged as an alternative to mobilisation alarms, given their uncertain effectiveness and negative consequences for patients and nurses. Much U.S. hospital research reflects a cost‐reduction paradigm aimed at replacing in‐person observers, a trend not seen internationally or in aged care. This research is relevant to decision‐makers considering technological options for falls prevention and to nurse leaders seeking insight into the appeal and apprehension surrounding video monitoring.

**Reporting Method:**

PRISMA‐ScR.

**Patient/Public Contribution:**

None.

## What Does This Paper Contribute to the Wider Global Clinical Community?

- This scoping review identified that the perceived effectiveness of remote video monitoring depends on the underlying rationale for its introduction—falls prevention, workforce optimisation, cost efficiency, or user experience.
- Technician‐based video monitoring systems are widespread in the United States, but this trend is not mirrored internationally or within residential aged care.
- There is interest in innovative remote video monitoring systems in residential aged care, yet research in this area remains sparse.

## 1 Introduction

Globally, falls continue to be a problem across hospitals and residential aged care (RAC). Both settings should continue to evaluate their fall prevention strategies to ensure they remain evidence‐based and effective to prevent the significant harms and costs associated with falls. Mobilisation alarms have historically been a common intervention within multifactorial fall prevention approaches in hospitals and RAC. These alarm devices come in a variety of types that alert staff when a person exits their bed or chair and may be at risk of falling. However, meta‐analyses (Morris et al. 2022) and a later published randomised controlled trial (RCT) (Pu et al. 2026) have shown that high use of alarms does not lead to reduced hospital falls. Additionally, dogma throughout RAC has highlighted the unwanted complications of alarms and fuelled an “anti‐alarm frenzy” and calls for “alarm‐free” policies in RAC (Steinberg 2018, 2). Alarms have widely documented drawbacks, including reduced patient/resident dignity, disruptive noise that disturbs sleep and increases agitation, feelings of restriction, and prompting reactive rather than proactive falls prevention practices (Hartmann et al. 2021; Stephen et al. 2025). These negative consequences have led to the pursuit of alternative devices to reduce the risk of falls for those people who require assistance with mobility but are unlikely or unable to request assistance.

Remote video monitoring systems have emerged as an alternative to traditional mobilisation alarms. Most systems fall into two broad categories. The first includes remote monitoring via standard monitoring screens that may or may not be watched continuously by staff. The second are emerging Artificial Intelligence (AI) enabled systems that use computer‐vision algorithms to detect fall risk behaviour and trigger alerts to staff so monitors do not require continuous staff observation. Another driver, specific to the United States (U.S.) hospital setting, was the Centers for Medicare and Medicaid Services (CMS) (2008) ruling that ceased reimbursing hospitals for costs associated with inpatient falls. It positioned falls as a direct financial liability thus driving strong financial incentives to focus on fall prevention interventions such as remote video monitoring. A further driver for remote monitoring implementation was the high costs and staffing shortages associated with in‐person observers frequently assigned to individual patients to reduce falls risk. A systematic review of in‐person observers in hospital settings identified eight video monitoring studies (Greeley et al. 2020). The evidence illustrated that video monitoring reduced the use of in‐person observers without increasing fall rates, and in some cases reduced falls. The review noted important limitations, including the absence of RCTs and the likelihood of publication bias.

The continuously monitored and AI enabled video systems have different implications for staffing, privacy, workflow and compatibility within hospital and RAC settings. Remote video monitoring may offer greater specificity than alarms because staff can remotely observe patient/resident behaviour without needing to be physically present. However, alarm fatigue and delayed responses can still occur, especially when staff are multitasking in dynamic, high‐stress environments. A review of 13 studies explored factors influencing acute hospital nurses' acceptance of remote monitoring for falls prevention (McNamara 2024). However, its conclusions specific to remote video monitoring were limited by the inclusion of studies evaluating mobilisation alarms, a falls‐detection device, and a systematic review of broader falls prevention health technology. Most included studies evaluated nurses' views as a secondary element in effectiveness studies with the level of nuance in participant responses limited. McNamara's review concluded three key factors influenced nurses' perceptions—effectiveness, ease of use and how staff felt supported by nursing leadership and hospital management. Our review builds on McNamara's review by focusing solely on remote video monitoring, incorporating additional hospital studies not captured in their review, and including RAC and the perspectives of patients and families. Understanding how remote video monitoring is used and perceived in each setting could help refine its role in falls prevention. Therefore, this scoping review examined the reasons for and methods of using remote video monitoring to prevent falls in hospital and RAC settings, and how staff, patients, residents, and families perceive its use and benefits.

## 2 Methods

### 2.1 Design

A systematic review was originally intended (CRD420251051480; Centre for Reviews and Dissemination 2025). However, the diversity of studies and our aim to map the ‘why’ and ‘how’ of video monitoring across various stakeholder perspectives and settings indicated that a scoping review approach was better suited. This enabled us to clarify the scope and breadth of the evidence base to guide future research priorities. The main protocol deviation was not conducting the risk of bias appraisal given our focus on describing the uptake of video monitoring and breadth of the evidence base, rather than making clinical recommendations based on the pooled data. The second departure from our initial plan was to report on video monitoring systems as a distinct, but related, category to mobilisation alarms. Remote video monitoring often serves purposes beyond falls prevention, and its implementation and processes differ substantially from alarms. Reporting these technologies separately enabled us to address the review question with more clarity. We followed the JBI guidance (Peters et al. 2020) to conduct this scoping review and reported it according to the PRISMA Extension for Scoping Reviews (Tricco et al. 2018).

### 2.2 Search Methods

An initial search of MEDLINE and CINAHL was performed to find studies related to the review question and identified free text keywords and controlled vocabulary to include in the full search strategy. The final search strategy was created with input from an academic librarian (Table S1). Databases were searched in July 2025 with key words and index terms adapted for each database. Databases included MEDLINE, Embase, Emcare, PsychINFO, CINAHL, AgeLine, Scopus and Clinical Trials registry. Studies were imported into Endnote 21 and duplicates removed prior to exporting into Covidence review software (2025). Reference lists of included studies and retrieved systematic reviews were screened for additional studies. One author was contacted regarding a linked qualitative study, though data was not yet available (Porock et al. 2024).

### 2.3 Inclusion and Exclusion Criteria

Eligibility criteria incorporated the Participant, Concept and Context framework (Table S2). Studies were included if they examined remote video monitoring where its primary purpose was to prevent falls. Video monitoring studies without the purpose of falls prevention were excluded. All designs and grey literature were eligible except for non‐research opinion papers and systematic reviews. There were no date or language restrictions. Participants included staff, patients, residents, or families. The concept was evidence relating to how and why remote video monitoring was used in falls prevention and attitudes towards its use.

Two independent reviewers (KS & RM) screened titles and abstracts and assessed full texts of potentially relevant studies against the inclusion criteria. Disagreements at the title/abstract and full‐text screening stages were settled by a third reviewer (DP).

### 2.4 Data Abstraction

A data‐charting template was developed in Microsoft Excel in advance and used to extract information from all relevant sections of each article. Data items included: author; year; country/setting; video monitoring type; methodology; sample; and key findings. We included data items related to study quality to offer contextual information about the strength of the evidence base to guide recommendations for future research priorities. One reviewer (KS) extracted data following a team planning meeting about how data would be extracted. The template was tested on five studies to ensure its suitability, and no significant revisions were required. The primary author (KS) completed the remainder of data extraction following the agreed upon template. Periodic checks were completed with the other authors.

### 2.5 Synthesis

Data related to study characteristics were presented descriptively. A convergent integrated approach combined quantitative and qualitative evidence. Quantitative data were converted to textual descriptions. However, some numerical results were retained to maintain accuracy and permit readers to understand the strength of evidence.

A narrative synthesis approach (Popay et al. 2006) was used to bring together the evidence about why and how remote video monitoring is used and stakeholders' attitudes towards its use. Patterns across the studies were described and relationships within the data explored to tell this story. A hybrid inductive‐deductive thematic synthesis approach was also used to allow a deeper exploration of attitudes towards the use and effectiveness of remote video monitoring (Thomas and Harden 2008). The primary reviewer (KS) conducted line‐by‐line inductive coding of each study. Coding was completed using structured tables in Microsoft Word with text colour‐coded to represent emerging codes and initial themes. Codes were refined and higher‐order analytical themes were developed in regular team meetings (Table S3). These themes were subsequently mapped deductively to the constructs of the Technology Acceptance Model (TAM). The TAM is commonly used in healthcare to help explain why users are willing or not willing to adopt a new technology (Davis 1989). Its core elements are perceived usefulness (the extent to which the technology is believed to enhance user performance) and perceived ease of use (the extent to which the person believes that using the technology is free from effort). These elements influence attitudes towards using the technology and actual technology adoption.

### 2.6 Rigour

The protocol was registered with PROSPERO given our initial intention to conduct a systematic review and complies with the PRISMA Extension for Scoping Reviews (PRISMA‐Scr) reporting guidelines. The different backgrounds of the authors (medical, nursing, physiotherapy, occupational therapy and speech therapy) and their sustained engagement with the data enhance the credibility of the review. Regular meetings discussing the review assisted confirmability, and the lead researcher kept a reflexive diary throughout the review process. Using both peer‐reviewed and grey literature enabled representation of a wider range of evidence.

## 3 Findings

### 3.1 Study Selection

Thirty‐five studies were included in this review (Figure 1). The initial search retrieved 2693 studies. Title and abstract screening reduced the number for full text review to 122. Application of the inclusion and exclusion criteria resulted in 35 studies selected for review (Figure 1).

**FIGURE 1:**
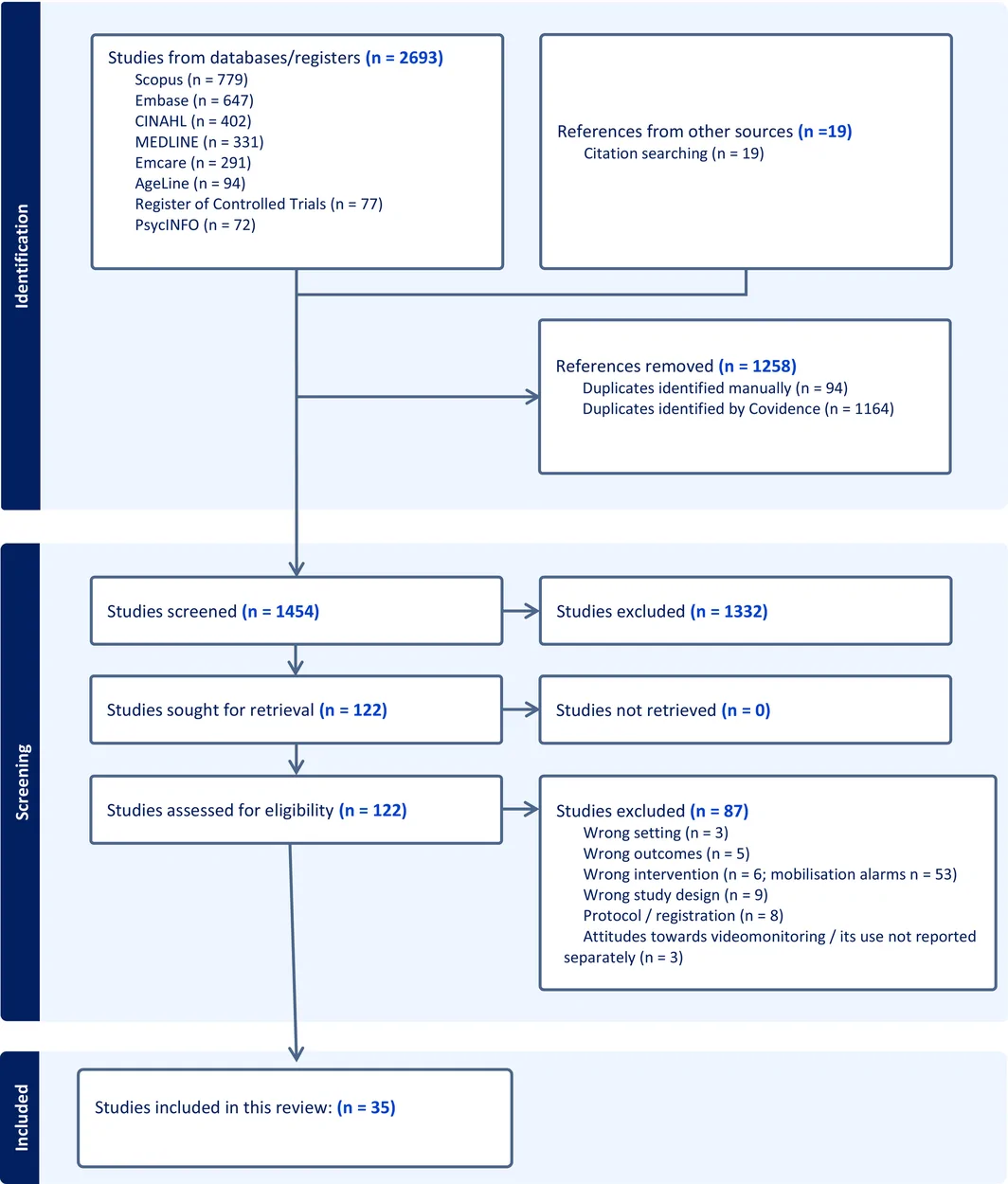
PRISMA diagram outlining data screening.

### 3.2 Characteristics of Included Studies

An overview of study characteristics is presented in Tables 1, 2, 3. Refer to Table S4 for a summary of the included studies' aims and key findings. The majority of research was hospital based (77%) and quantitative (60%). The volume of publications increased steadily over time with approximately half of the studies published within the last 5 years. Twenty‐two studies examined newly implemented remote video monitoring and 13 studies explored perceptions of it embedded within routine care. An important characteristic of this literature is the stark geographical divide between care settings. The U.S. dominates hospital‐based research, but its representation in RAC is almost entirely absent. Of the 35 included studies, 24 (69%) originated from the U.S., with all focused exclusively on the hospital environment except Vandenberg et al. (2017), which compared traditional alarms in a U.S. RAC home with more advanced monitoring systems in a Dutch RAC home. Only four hospital‐based studies were conducted outside the U.S. (Fitzpatrick 2019; Geil Kollerup et al. 2021; Woltsche et al. 2022; Zubrinic et al. 2023), also indicating a gap exists in international hospital remote video monitoring research.

**TABLE 1:** Characteristics of hospital studies (*n* = 27).

Category	Subcategory	Hospital studies *n* (%)
Remote video monitoring type	Remote technician‐based video monitoring systems	17 (63%)
	Nurse‐observed video monitoring system without alerts	3 (11%)
	Automated video monitoring systems that generate alerts	5 (19%)
	Video monitoring type not specified	0
	Video monitoring and traditional pressure sensitive	2 (6%)
Topic focus	Staff attitudes	22 (81%)
	Patient, resident or family attitudes	5 (19%)
	How remote video monitoring is used	9 (33%)
Year of publication	2011–2015	4 (15%)
	2016–2020	10 (37%)
	2021–2025	13 (48%)
Country	United States	23 (85%)
	Canada	1 (4%)
	Australia	1 (4%)
	United Kingdom	1 (4%)
	Netherlands	0
	Germany	0
	Denmark	1 (4%)
	Sweden	0
	Japan	0
Study design	Quantitative	20 (74%)
	Qualitative	4 (15%)
	Mixed methods	3 (11%)

**TABLE 2:** Characteristics of RAC studies (*n* = 8).

Category	Subcategory	RAC studies *n* (%)
Remote video monitoring type	Remote technician‐based video monitoring systems	0
	Nurse‐observed video monitoring system without alerts	4 (50%)
	Automated video monitoring systems that generate alerts	2 (25%)
	Video monitoring type not specified	2 (25%)
	Video monitoring and traditional pressure sensitive	4 (50%)
Topic focus	Staff attitudes	8 (100%)
	Patient, resident or family attitudes	4 (50%)
	How remote video monitoring is used	0
Year of publication	2011–2015	2 (25%)
	2016–2020	3 (38%)
	2021–2025	3 (38%)
Country	United States	1 (13%)
	Canada	0
	Australia	2 (25%)
	United Kingdom	0
	Netherlands	3 (35%)
	Germany	1 (13%)
	Denmark	0
	Sweden	1 (13%)
	Japan	1 (13%)
Study design	Quantitative	1 (13%)
	Qualitative	3 (35%)
	Mixed methods	4 (50%)

**TABLE 3:** Mapping of study level characteristics across hospital and RAC settings.

First author	Year	Setting		Study design			Country	Study focus			Remote video monitoring type				
		Hospital	RAC	Quantitative	Qualitative	Mixed methods		Staff attitude	Patient Family attitude	How video monitoring is used	Remote technician‐based video monitoring systems	Automated video monitoring systems that generate alerts	Nurse‐observed video monitoring system without alerts	Video monitoring type not specified	Video monitoring & pressure sensitive alarm
Abbe	2022	✓		✓			U.S.	✓			✓				
Allen	2023	✓		✓			U.S.	✓				✓			
Baez	2022	✓		✓			U.S.	✓					✓		
Barnett	2020	✓		✓			U.S.	✓			✓				
Borda	2020		✓			✓	Australia	✓				✓			
Burtson	2015	✓		✓			U.S.			✓	✓				
Cournan	2018	✓		✓			U.S.	✓			✓				
Emilsson	2023		✓		✓		Sweden	✓					✓		✓
Ergai	2024	✓		✓			U.S.	✓			✓				
Fenton	2016	✓			✓		U.S.	✓				✓			
Fischer	2024		✓			✓	Germany	✓	✓					✓	
Fitzpatrick	2019	✓		✓			United Kingdom	✓				✓			
G.Kollerup	2021	✓				✓	Denmark	✓					✓		
Hardin	2013	✓				✓	U.S.	✓				✓			✓
Hawksworth	2022	✓			✓		U.S.	✓			✓				
Hebb	2021	✓		✓			U.S.	✓		✓	✓				
Hu	2024	✓			✓		U.S.		✓		✓				
Iio	2016		✓			✓	Japan	✓	✓			✓			✓
Jeffers	2013	✓		✓			U.S.	✓		✓	✓				
Klymko	2016	✓			✓		U.S.	✓			✓				
Latham	2023	✓		✓			U.S.	✓	✓		✓				
McCurley	2014	✓				✓	U.S.	✓	✓		✓				
Nwaise	2017	✓		✓			U.S.	✓			✓				
Porock	2024		✓	✓			Australia	✓	✓				✓		
Purvis	2018	✓		✓			U.S.	✓		✓					
Quigley	2022	✓		✓			U.S.			✓					✓
Rivera	2023	✓		✓			U.S.	✓				✓			
Sand‐Jecklin	2019	✓		✓			U.S.	✓	✓		✓				
Sand‐Jecklin	2016	✓		✓			U.S.			✓	✓				
Vandenberg	2017		✓		✓		U.S./Netherlands	✓					✓		
Vortuba	2016	✓		✓			U.S.			✓	✓				
Woltshe	2022	✓		✓			Australia	✓		✓			✓		
Zubrinic	2023	✓		✓			Canada	✓	✓	✓	✓				
Zwijsen	2012		✓		✓		Netherlands	✓					✓		✓
Zwijsen	2011		✓			✓	Netherlands	✓	✓					✓	✓

## 4 Types of Remote Video Monitoring

### 4.1 Remote Technician‐Based Video Monitoring Systems

Remote technician‐based video monitoring systems were the most commonly examined systems (63%). All studies originated in the U.S., except for one Canadian study (Zubrinic et al. 2023). These systems involve cameras with two‐way speakers permanently installed in patients' rooms or placed on mobile carts. Video monitoring technicians are located remotely (usually within the hospital, but separate to the wards) and they observe multiple high‐risk patients through live video feeds. Technicians simultaneously monitor between 6 (Purvis et al. 2018) and 15 (Ergai et al. 2023) patients. Hospitals implementing these systems frequently cited staff shortages as a key driver. Some hospitals hired new technicians, while others re‐assigned inpatient observers into technician roles. Notably, no studies reported that adding technicians resulted in a net increase in overall staffing levels.

Some systems allow technicians to deliver pre‐recorded prompts in the patient's preferred language (Abbe and O'Keeffe 2022), and all were reported to allow direct communication with the patient and nursing team, including activation of a STAT alarm for urgent responses. One study was an exception (Baez and Karl 2022). They introduced a remote video system but did not include a dedicated technician to monitor the videos. Hence it was the nurses' responsibility to provide intermittent surveillance via the monitors, and they could verbally communicate with the patient as required. No RAC studies evaluated systems that incorporated dedicated monitoring technicians.

Various studies outlined the uptake of technician‐based video monitoring – some suggested it was underutilised (Nwaise 2017), whilst others described the need for waitlists due to its high demand (Abbe and O'Keeffe 2022; Burtson and Vento 2015). In a survey of 55 nurses, 26% indicated they utilised video monitoring daily (Hebb et al. 2021), and mobile video device units were in use 90%–95% of the time (Abbe and O'Keeffe 2022). Another study across five hospitals reported using remote video monitoring for 1953 patients over a two‐year period (Zubrinic et al. 2023). Only two studies detailed the length of time video monitoring was used for individual patients. Acute patients were monitored for an average of 2.5–3.3 days, and during each shift required 1.2 to 10 verbal technician redirections and 7–11 nurse alert calls (Sand‐Jecklin et al. 2016; Vortuba et al. 2016). In a rehabilitation brain injury unit, technicians averaged 95 interventions per shift, but the study did not differentiate these by patient or by intervention type for example, verbal redirection or nurse‐alert call (Cournan et al. 2018).

### 4.2 Automated Video Monitoring Systems That Generate Alerts

Automated video monitoring systems represented 20% of included studies and were characterised as emerging, technologically advanced approaches. They were limited to small pilot evaluations, with their large‐scale implementation across either setting yet to be established. Depth sensor data is processed by computer‐vision algorithms, to detect movements consistent with a patient attempting to exit their bed or chair and sends an alert to nurses via their mobile devices or a central monitor at the nurses' station (Abbe and O'Keeffe 2022; Fitzpatrick 2019). In efforts to partially preserve patient privacy, generated alerts are de‐identified live views of patients as skeletal stick‐figure models (Borda et al. 2020; Rivera et al. 2023) or blurred silhouettes (Iio et al. 2016; Fenton et al. 2016). One study incorporated webcams with range sensors described as “virtual bed rails.” When patients crossed the motion‐sensitive borders, the system generated visual and audible alerts at the nurses' station (Hardin et al. 2013).

### 4.3 Nurse‐Observed Video Monitoring Systems Without Alerts

The nurse‐observed video monitoring systems were more commonly evaluated in RAC. They use passive video feeds that are only intermittently observed by existing nursing staff as part of routine duties, without dedicated monitoring technicians or automated alerts. Staff must physically attend the patient to mitigate falls risk rather than verbally redirect them remotely. Half of the RAC‐based research explored perspectives on this type of system that used stationary monitoring screens (Emilsson et al. 2023; Porock et al. 2024; Vandenberg et al. 2017; Zwijsen et al. 2012). Two hospital‐based pilot studies examined portable video monitors carried by nurses enabling them to observe (Geil Kollerup et al. 2021), and/or hear patient behaviour (Woltsche et al. 2022) while performing routine duties.

The proportion of RAC homes using remote video monitoring and the average number of residents monitored per home remain unknown, as no studies investigated this. Porock et al. (2024) indicated that most family members surveyed (85% of 74) stated they had not installed a camera in their resident's room, although 38% had considered it. This suggests a need for video monitoring to be actively discussed with residents and families, particularly given staff reported greater awareness of its use than families. Only one study described the number of residents being monitored. Emilsson et al. (2023) reported 44 out of 50 residents used their newly implemented video monitoring.

## 5 Attitudes Among Staff, Patients, Residents and Family Members Towards Remote Video Monitoring

The majority of studies focused exclusively on staff perspectives or a combination of staff and family perspectives. Patient and resident (both those monitored as well as those present but not monitored) perspectives were rarely evaluated. Only one study focused exclusively on the patient perspective, and this was limited to a conference abstract with the patient experience interpreted and presented through the lens of the clinician‐researcher rather than a direct patient‐led account (Hu et al. 2024).

Six themes were constructed across hospital and RAC studies pertaining to attitudes towards remote video monitoring. These themes represented contextual determinants of the Technology Acceptance Model's core elements (Figure 2). The underlying motivation to implement remote video monitoring shaped whether it was considered effective. Perceived effectiveness and workload influenced perceived usefulness, while device knowledge and workload informed perceived ease of use. Ethical and liability concerns moderated how usefulness and ease of use were interpreted, which ultimately had the potential to influence the uptake of remote video monitoring in practice. Refer to Table S5 for key quotes depicting themes.

**FIGURE 2:**
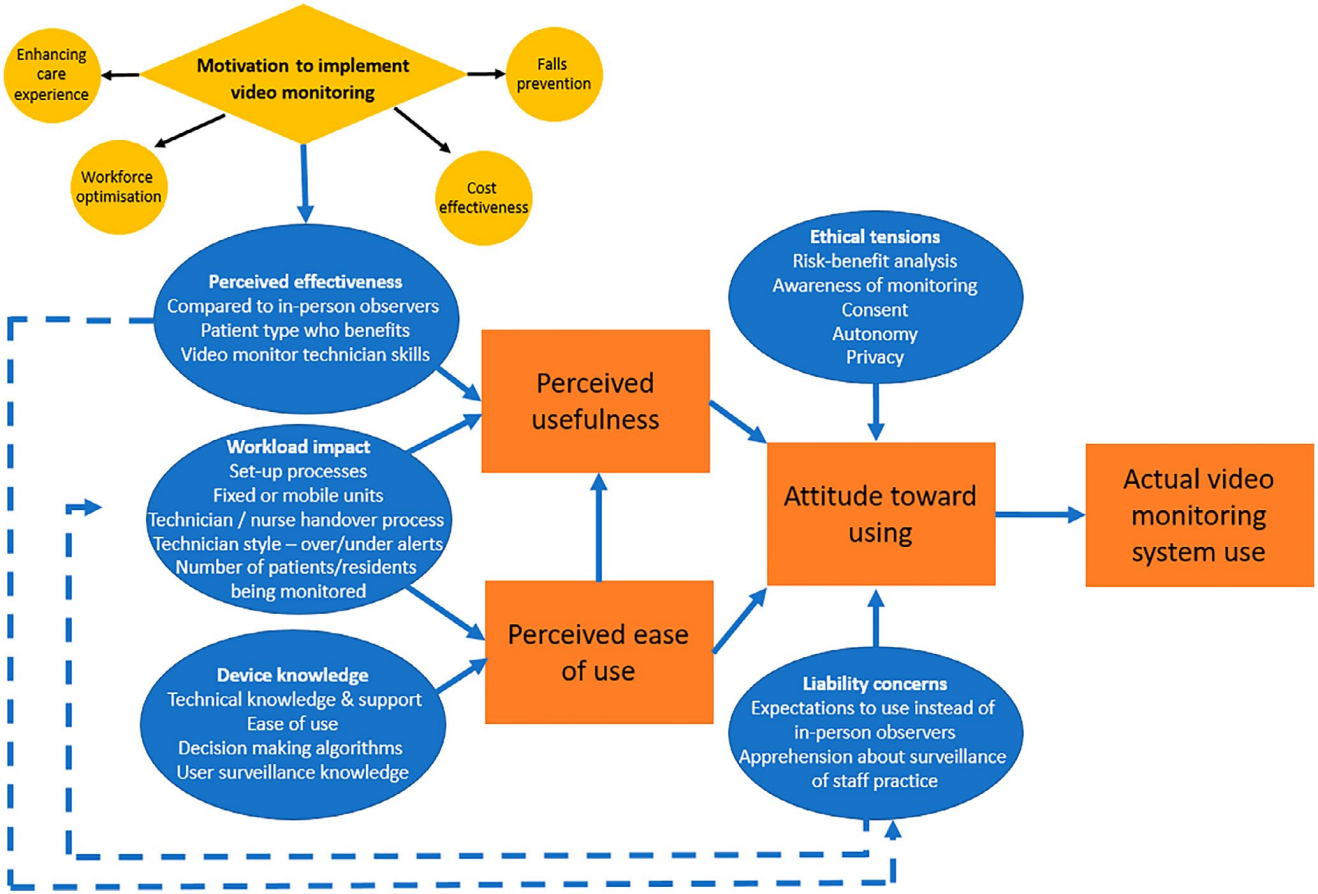
Themes shaping attitudes towards remote video monitoring mapped to the Technology Acceptance Model.

### 5.1 Implementation Motivations for Remote Video Monitoring

Motivations for evaluating remote video monitoring systems varied considerably across the literature, shaping how “success” and “effectiveness” were ultimately defined (Table 4). Some studies reflected a single dominant rationale, whilst others incorporated multiple motivations within the same evaluation. Differing motivations influenced stakeholder perspectives differently. Executives and health service decision makers tended to frame benefits as aligned with organisational drivers that prompted adoption, such as efficient resource use, whereas patients, families, nurses, and monitoring technicians mostly described attitudes based on their personal, day‐to‐day experiences of the system.

**TABLE 4:** Motivations for evaluating remote video monitoring systems and their implications for perceived effectiveness.

Underlying motivation/research paradigm	Problem prompting adoption	Primary outcome used to judge effectiveness	How success is framed
Falls prevention	Limited effectiveness of traditional mobilisation alarms; need for alternative fall prevention strategies	Reduction in falls	If fall rates decreased compared with baseline or usual care
Staffing efficiency/workforce optimisation	High use of in‐person observers; diversion of nurses from routine duties to in‐person observer roles	Maintenance of fall rates while reducing in‐person observer use	If nurses could return to direct care without an increase in falls
Cost‐effectiveness	High labour costs associated with in‐person observers; interest in lower‐cost monitoring models	Comparative cost savings between remote technician‐based monitoring and in‐person observers	If expenditure was reduced while maintaining acceptable safety outcomes
Experience‐based/frontline perspectives	Day‐to‐day impact of monitoring on workload, privacy, and patient experience	Perceived acceptability and useability	Interpreted through lived experience rather than organisational benchmarks

Although all included studies implemented remote video monitoring primarily for falls prevention, authors frequently noted additional, opportunistic benefits arising from system use. These included identifying seizures (Jeffers et al. 2013; Sand‐Jecklin et al. 2019; Vortuba et al. 2016), high risk behaviours such as self‐harm, drug diversion/use (Sand‐Jecklin et al. 2019), wandering or elopement (Latham 2023, Sand‐Jecklin et al. 2019; Vortuba et al. 2016), and pulling out medical devices (Purvis et al. 2018; Sand‐Jecklin et al. 2019; Vortuba et al. 2016; Zubrinic et al. 2023). Other video monitoring features were not framed specifically as falls prevention strategies but may be argued as indirectly related. One AI computer‐vision system detected both patient and staff position in the room and displayed visual reminders on staff handheld devices of the time since last rounding, serving as a “clinical nudge to improve compliance” with hourly rounding protocols (Rivera et al. 2023, 25). Another ancillary benefit relevant to falls prevention was the reduction in the amount of physical checks required, which was thought to promote patient sleep (Geil Kollerup et al. 2021).

### 5.2 Perceived Effectiveness

#### 5.2.1 Perceived Suitability for Patients and Residents for Remote Video Monitoring

Only the literature on remote technician‐based video monitoring consistently described eligibility criteria for patients considered likely to benefit from monitoring. Studies of automated or nurse‐observed systems were generally less structured and did not outline such criteria. Across the studies, patients with reduced insight, impulsive behaviours, and/or at a high risk of falls were deemed appropriate. Common clinical presentations included dementia, delirium, day‐one postoperative status, psychiatric illness, stroke, alcohol withdrawal, or impaired mobility (Hardin et al. 2013). Pre‐existing auditory hallucinations were listed as an exclusion criterion (Zubrinic et al. 2023), presumably to avoid misinterpreting the monitoring technician's voice.

A perceived advantage over traditional mobilisation alarms was technicians providing verbal direction to patients to reassure or remind them to wait for the nurse (McCurley and Pittman 2014). However, a conundrum was present in that whilst confused patients were thought to benefit from remote video monitoring, they had to be redirectable and follow verbal commands from the technician. If unable to do this, either due to poor cognition, hearing, or non‐English speaking background, then patients were often considered ineligible (Sand‐Jecklin et al. 2019). Other times, patients who were startled by the “intercom voice” and unable to follow the technician's directions were not excluded from monitoring, but direct paging of nurses occurred rather than technician‐patient communication (Klymko et al. 2016).

The study which examined portable video monitors specifically targeted restless and confused patients and did not rely on remote redirection. Instead, video feeds were streamed to nurses' mobiles, and they attended the patient in person when needed (Geil Kollerup et al. 2021). In this study, 94% of nurses agreed that video assisted observation should be an option in acute care. However, they also highlighted that effectiveness was linked to their ability at the time to immediately attend when they observed fall risk behaviour via their device. This is also true of remote technician‐based monitoring—nurses still experience difficulties attending to technician alerts when busy with other patients or critical tasks when the technician alert is raised (Zubrinic et al. 2023).

Other reasons where patients were deemed unsuitable for technician‐based video monitoring was if they required such close monitoring that the technician was unable to adequately watch other patients, the patient was too fast for staff to respond to alarms generated by the technician, or the patient consistently required more than three verbal redirections or staff alarms within 30 min (Hawksworth et al. 2022; Purvis et al. 2018; Quigley et al. 2022). Patients requiring too many redirections or alarms were allocated an in‐person observer instead. Whilst not explicitly stated, this presumably freed up both technician and nursing staff to direct their attention and care to other patients.

One RAC‐based feasibility study's strict patient eligibility criteria stood out from other studies (Borda et al. 2020). This study specifically targeted patients who had at least two falls in the past 6 months and were able to walk independently or with staff assistance. It excluded those with no falls history, were palliative, bed bound, or required a hoist for all transfers. It might be that those patients excluded were still at risk of falls, but the labour‐intensive installation process and early‐stage testing prompted the researchers to develop more stringent criteria to minimise participants whilst in the prototype phase.

#### 5.2.2 Perceived Effectiveness Across Stakeholder Groups

Patients and families were mentioned briefly in several studies (Geil Kollerup et al. 2021; McCurley and Pittman 2014; Zubrinic et al. 2023), with general comments suggesting they viewed remote video monitoring as effective and felt reassured that staff could observe patients and respond quickly when not present. However, these assertions were either based on single case reports or lacked accompanying data to substantiate these claims. Sand‐Jecklin et al. (2019) was the exception. In their survey of 52 patients and family members, 22 respondents (42%) reported remote video monitoring protected patients' safety “very well.” Only three respondents (6%) explicitly stated concerns about remote video monitoring.

Nurses consistently described remote video monitoring as effective for preventing falls, primarily due to enabling a “second pair of eyes” (Jeffers et al. 2013, 303; Hawksworth et al. 2022). This was thought to be valuable in dynamic hospital settings where patients were at increased risk of falling due to environment factors (e.g., loose cords, wet floors, objects out of reach, room changes), process‐based factors (e.g., shift changes, meal times, sleep disruptions), and patient‐based factors (e.g., incontinence, pain, anxiety; Klymko et al. 2016). Remote video monitoring was considered particularly effective for patients far away from the nurses' station in low visibility areas (Hardin et al. 2013; Zubrinic et al. 2023).

U.S. hospital nurses often preferred in‐person observers, despite remote video monitoring still regarded as effective and often introduced to reduce reliance on in‐person observers (Barnett et al. 2020; Hebb et al. 2021). This preference aligns with the constructs of the TAM. Setting up and responding to video monitoring alerts required additional effort, indicating lower perceived “ease of use” compared to the use of in‐person observers. In‐person observers may also be considered as having higher “perceived usefulness” because they can intervene immediately to prevent a fall, whereas remote video monitoring still requires nurses to physically respond to alerts at times. Staff acknowledged remote video monitoring contributed to patient safety, although this was the lowest rated domain compared to attitudes towards use, perceived usefulness, intention to use, and perceived ease of use (Ergai et al. 2023). Hospital managers introducing remote video monitoring found staff were initially resistant, wedded to the idea of in‐person observers being the only effective intervention, but perceptions improved over time (Fitzpatrick 2019; Allen et al. 2023).

Residents and families within RAC appeared willing to consider innovative monitoring solutions to prevent falls (Borda et al. 2020; Fischer et al. 2024; Iio et al. 2016). Some topics were emphasised in RAC, but not raised in hospital‐based research, such as potential dual purposes of remote video monitoring to detect falls and/or prevent falls and if it was effective to monitor public and/or private areas. Residents valued the ability to change video functions to increase monitoring capacity (without or with constant observation) according to what level of care they required (Iio et al. 2016). Psychogeriatric RAC staff, who used less intrusive monitoring (infra‐red sensors) for private areas and limited video monitoring to public areas only, wished for expansion of video monitoring into private areas (Vandenberg et al. 2017). Staff and residents perceived remote video monitoring gave them more security and was more effective than traditional mobilisation alarms (Iio et al. 2016), yet it was not considered an effective substitute for human presence (Emilsson et al. 2023). Similar to hospitals, remote video monitoring effectiveness in RAC was perceived to depend on how timely staff can respond (Borda et al. 2020).

### 5.3 Workload Impact

The ability to start remote video monitoring immediately, rather than spend time sourcing in‐person observers, was considered advantageous (McCurley and Pittman 2014). However, adding or removing a patient from remote video monitoring was occasionally viewed as burdensome (Hardin et al. 2013; Cournan et al. 2018). One study explored different professional groups' perspectives to determine if they should pursue automated video monitoring (Fenton et al. 2016). Therapists were the only group to express concerns that video monitoring might affect personal productivity. This may be because responding to video generated alerts fell beyond their traditional clinical role.

Most hospitals implemented clearly defined criteria and algorithms to support staff in deciding which patients received remote video monitoring and regular reassessment of its suitability or discontinuation. RAC did not appear to have comparable formal processes. The structured hospital processes such as standardised handovers and documentation of monitoring technician interactions, whilst good for quality of care, were viewed as cumbersome and time consuming (Hawksworth et al. 2022). The technician's monitoring style potentially impacted workload demands. Concerns were raised about technicians themselves being overloaded, as well as nurses partnered with hypervigilant technicians who frequently sounded alerts (Hawksworth et al. 2022). Among 55 surveyed nurses, 40% were dissatisfied with technicians' communication about patients requiring assistance; however, it is unclear whether this dissatisfaction stemmed from technicians who over‐ or under‐requested nursing assistance (Hebb et al. 2021). Examples were provided where retraining targeted technicians to increase the frequency they redirected patients directly via the microphone (Vortuba et al. 2016).

Most hospitals implemented live video monitoring systems without recording capabilities to alleviate consent and privacy concerns. However, Fitzpatrick (2019) evaluated video with recording, and staff thought it benefitted both patient care and workload. The hospital's policy was to assume patients hit their head in unwitnessed falls and required staff to conduct 15 min observations. Replaying video images meant staff could confirm if patients hit their head or not. If a head strike did not occur, staff did not have to disturb patients unnecessarily via frequent observations and were able to redirect this time to other important tasks.

Technician‐based monitoring does not feature in the RAC literature. A potential challenge in rolling out hospital remote video monitoring was safely discharging patients who relied on it back to RAC homes without the same monitoring (Purvis et al. 2018). Introducing nurse‐observed video monitoring into RAC settings came with significant technical challenges and increased staff workload, yet staff still considered it worthwhile (Borda et al. 2020; Emilsson et al. 2023). Perceived workload benefits included fostering staff teamwork and the ability to prioritise responses based on camera images (Iio et al. 2016; Emilsson et al. 2023). Avoiding unnecessary physical bedside visits meant residents were disturbed less overnight, which staff believed improved sleep and behaviour, indirectly reducing workload (Emilsson et al. 2023; Fischer et al. 2024). Families reported concerns that remote video monitoring may replace much needed staff‐resident contact in an attempt to reduce workload (Porock et al. 2024). However, findings from a small pilot study investigating a depth‐sensor‐based video system suggested it did not alter staff's relationship or frequency of resident contact (Borda et al. 2020).

### 5.4 Applied Device Knowledge

Some shortfalls were apparent in device training for staff. Occasionally, staff training occurred too early, before the video infrastructure was installed. Staff could not yet appreciate how their work patterns might change once video monitoring was in place (Borda et al. 2020; Emilsson et al. 2023). Staff perceived there was insufficient support from suppliers and a sense that vendor information received did not in reality work. This required staff to troubleshoot problems, relying on colleagues with technical knowledge to upskill peers (Emilsson et al. 2023).

Education regarding remote video monitoring processes appeared more prominent in hospital‐based studies. There were three main purposes of education and training. First, to change culture by educating nurses how remote video monitoring could be a safe alternative to in‐person observers (Burtson and Vento 2015). This education involved structured policies, decision‐making algorithms, and highlighting the ‘trickle‐down effect’ of costly in‐person observer overuse (Hawksworth et al. 2022). Second, staff needed training in actual device set‐up and use. Finally, education addressed how technicians and nurses collaborated to use video monitoring effectively, including developing technician's skills to recognise impending falls and take the most appropriate action or alert (Quigley et al. 2022; Zubrinic et al. 2023). Sometimes video vendors provided organisations resources such as technician job descriptions, education tools and protocols, which organisational leaders then customised (McCurley and Pittman 2014). Educating nurses about video functions and protocols promoted user acceptance and confidence (Hawksworth et al. 2022; Nwaise 2017).

Technicians' skills to detect fall risk behaviour were sometimes questioned (Hawksworth et al. 2022), and hospital environments posed constant patient, structural and process challenges to remote video monitoring (Klymko et al. 2016). Competency evaluation tools were created to validate technician knowledge (Jeffers et al. 2013; Sand‐Jecklin et al. 2019). Four studies sought technicians' perspectives (Klymko et al. 2016; Latham 2023; McCurley and Pittman 2014; Sand‐Jecklin et al. 2019). Some technicians expressed feeling ill‐equipped to communicate with nurses, were unfamiliar with medical terminology and felt uncomfortable monitoring some patients (Sand‐Jecklin et al. 2019). A variety of supportive technician training approaches were described, ranging from technical support (camera zooming, voice activation, privacy screens) to job simulations, in‐person observer support, flow charts, troubleshooting, and cleaning instructions (Jeffers et al. 2013; Vortuba et al. 2016; Cournan et al. 2018; Purvis et al. 2018; Ergai et al. 2023).

Nurse knowledge was usually supported via protocols and decision‐making tools (Cournan et al. 2018; Purvis et al. 2018; Ergai et al. 2023), but training appeared less comprehensive than technician training. In one study of 106 nurses, 55 (52%) reported not receiving education on remote video monitoring (Ergai et al. 2023), and in another study, only 29 of the 55 nurses surveyed (54%) reported feeling satisfied with the education received (Hebb et al. 2021). Despite this, nurses still rated video monitoring patient selection criteria and protocols easy to understand (Barnett et al. 2020; Ergai et al. 2023), thus suggesting that perhaps additional more nuanced education was required. Educational videos and hospital intranet resources were suggested, or implemented, for staff to access anytime to increase reach across different shifts to optimise video adoption and sustainment (Purvis et al. 2018; Woltsche et al. 2022). A need to retrain staff when processes were suboptimal was also highlighted (Vortuba et al. 2016).

Patient and family education was rarely addressed. One hospital outlined an additional role—that of a remote video monitor technician facility liaison (Ergai et al. 2023). Their role acted as a conduit between patient, technician and nurse, and they set up/removed camera equipment. Occasionally, there were procedures where technicians visited patients' rooms to explain video monitoring's role in keeping them safe (McCurley and Pittman 2014). A Patient and Family Advisory Council emphasised that patient/family education handouts should include information that technicians are trained nursing assistants and there is no video recording to promote its acceptance (Purvis et al. 2018). In the survey of 52 patients and family members, 33% reported remote video monitoring was not explained, and 44% reported privacy protection was not explained at all (Sand‐Jecklin et al. 2019).

### 5.5 Liability Concerns

For hospital administrators, the primary driver to adopt remote video monitoring appeared to be its cost‐effectiveness compared to in‐person observers, rather than concerns about mitigating potential liability from patient falls. In fact, some frontline staff felt pressured to use remote video monitoring by managers while simultaneously fearing increased liability because they believed in‐person observers were the most effective fall prevention method. Nurses highlighted the importance of balancing adherence to guidelines to use remote video monitoring and valuing independent assessment of what was required to keep patients safe (Hawksworth et al. 2022). Procedures were developed to appease apprehensive staff where remote video monitoring was initially used in conjunction with an in‐person observer to confirm its sufficiency to keep patients safe (Burtson and Vento 2015; Purvis et al. 2018).

Hospital leaders reported potential advantages of remote video monitoring were to audit capability and compliance (Fenton et al. 2016; Rivera et al. 2023). This potentially triggered liability concerns if staff were required to defend themselves for any misconduct inadvertently captured via video. Fear of data abuse (Fischer et al. 2024), discomfort, and feelings of being “watched or judged” were raised by some RAC (Vandenberg et al. 2017; Emilsson et al. 2023; Porock et al. 2024) and hospital staff (Hardin et al. 2013).

### 5.6 Ethical Tensions

Privacy concerns were frequently addressed in the literature across hospital and RAC. Three video features allayed privacy concerns in hospitals: no recording capability, temporary privacy screens, and non‐identifiable images for depth‐based monitoring. Most hospital systems had live feeds of standard camera images. Sometimes disciplines other than nursing expressed privacy concerns for themselves as well as patients. Notably, doctors felt video monitoring was potentially more intrusive to themselves than patients (Fenton et al. 2016). It was suggested that staff expressing concerns be provided with information and opportunities to observe remote video monitoring in practice to reassure them (Purvis et al. 2018). Privacy screens were common, allowing nurses or patients to request that technicians turn off the video during personal care. Patients were aware of the privacy mode via an indicator light (McCurley and Pittman 2014). In an online survey (sample size not reported), 91% of staff perceived remote video monitoring as unobtrusive, and patients and families reported it less intrusive than in‐person observers (Cournan et al. 2018).

Hospitals varied whether patient or family consent for remote video monitoring was required. Scepticism was expressed about the process of obtaining informed consent given patients eligible for remote video monitoring generally were unable to provide informed consent themselves. In a small feasibility study, 39 out of the 44 participants required informed consent to be provided by a relative (Geil Kollerup et al. 2021). Hardin et al. (2013) reported low consent rates, citing privacy concerns, whereas hospitals where remote video monitoring was standard care and required active refusal had much lower refusal rates. Sometimes efforts were made to direct camera angles away from patient faces to maintain privacy, but then this limited the extra cues that observing facial features provided, such as emotional states or attempts at communication (Geil Kollerup et al. 2021). There was also the issue of patients in shared rooms who did not require monitoring but were inadvertently captured on camera (Hawksworth et al. 2022). One case highlighted the harmful impact of an inflexible hospital policy that prohibited a patient‐requested camera covering. Although the camera was inactive, the patient's mistrust of it damaged rapport with staff and they threatened to discharge against medical advice (Hu et al. 2024).

Unlike hospital, often RAC cameras had recording capability, and individual informed consent was obtained from the resident or family (Emilsson et al. 2023; Vandenberg et al. 2017). Cognitive impairment or ‘usual care’ were not reasons to exclude seeking consent. “Individual continuous assessment” of consent was recommended for residents who had forgotten the camera's purpose but how this was practically implemented was not addressed (Emilsson et al. 2023, 10).

Another area specific to RAC was whether public or private areas were monitored. Staff perspectives on cameras in bedrooms varied—some supported it (Vandenberg et al. 2017), whilst others were concerned (Porock et al. 2024). Remote video monitoring was viewed as functionally equivalent to restraint if it restricted privacy and autonomy. Nurses were more likely to classify video observation as a restraint than bed alarms (Zwijsen et al. 2011). Families had less concerns than staff regarding monitoring in private areas, particularly if residents had dementia.

The juggle of benefits and risks of remote video monitoring according to individual needs and preferences was evident in RAC but rarely in hospital. Some residents indicated they were not comfortable with cameras placed in their rooms (Emilsson et al. 2023), whilst others wanted monitoring all the time (Iio et al. 2016). The right balance is required between increased safety and decreased privacy. Too much surveillance was destructive to a ‘home like’ atmosphere (Porock et al. 2024). Alternatively, remote video monitoring was felt to facilitate respectful care for those residents who valued their privacy, replacing the need for regular physical monitoring which was considered intrusive. Depth‐based systems that displayed stick‐figure or silhouette representations were perceived as good for privacy and confidentiality (Iio et al. 2016; Borda et al. 2020).

Data abuse concerns were mentioned by staff and family (Fischer et al. 2024). The importance of clear RAC guidelines to avoid privacy invasion and misuse was emphasised, including guidance on camera placement, procedures for accessing recordings, and limits on viewing duration (Emilsson et al. 2023). Also important was communication of how actively video systems were monitored. Families assumed the video feed was continuously monitored and that staff could respond in real time, which was not always the case and may have falsely reassured them about their relative's safety (Porock et al. 2024).

## 6 Discussion

This scoping review explored how remote video monitoring is used for falls prevention and how it is perceived within hospital and RAC. More than three quarters of the included studies (27 of the 35; 77%) were conducted in hospitals, and of these, 23 were conducted in the U.S. The CMS ruling of non‐reimbursement for in‐hospital falls (CMS 2008) may be driving U.S. practice in a direction that is different to the rest of the world. In this context, in‐person observers became a standard falls prevention approach, and remote video monitoring technicians were introduced primarily in a cost‐minimisation paradigm (reduce costs of in‐person observers without increasing falls relative to usual care). This contrasts with other countries, where the research paradigm is that remote video monitoring introduction would lead to increased costs, and would therefore need to be justified by reducing falls compared to usual care. Whilst falls are problematic across hospitals and RAC, only 8 of the 35 (23%) studies were RAC‐based. This imbalance may partly reflect the disproportionate volume of hospital‐based research, given hospitals typically have more resources and research capacity than RAC. Nevertheless, the pattern suggests that researchers have historically prioritised hospital settings, leaving RAC underrepresented in the literature evaluating innovative fall prevention approaches, including remote video monitoring—a critical evidence gap.

No technician‐based video monitoring systems were evaluated in RAC, whereas 17 studies evaluated this model in hospitals. The reason for this is unclear. Hospitals have realigned many in‐person observer roles to remote technician‐based video monitoring so that multiple patients can be observed simultaneously. RAC settings have not traditionally had access to in‐person observers and already operate under such tight staffing ratios and financial constraints. Therefore, securing funding for an additional staff member to adopt a technician role is likely difficult. An additional barrier may be the ethical issues surrounding monitoring people in their own homes. However, some older people expressed that, although they did not currently require high levels of care, they were open to constant observation if their needs escalated (Iio et al. 2016). This willingness, combined with family expectations that continuous video monitoring is actively observed by staff (Porock et al. 2024), suggests that feasibility research evaluating the effectiveness and acceptability of a technician‐based model in RAC could be warranted.

RAC has a high proportion of residents at high risk of falls that also have cognitive and hearing impairments which make it difficult for them to follow commands. These comorbidities are potential barriers to effective technician intervention (Sand‐Jecklin et al. 2019). In hospital, technicians were encouraged to increase attempts to verbally redirect patients rather than alert nurses (Vortuba et al. 2016), an approach that assumes patients can comprehend technicians' verbal redirections. Some studies reported that technician‐based monitoring successfully reduced the need for in‐person observers and maintained (Purvis et al. 2018) or reduced falls (Jeffers et al. 2013; Quigley et al. 2022). This may explain why hospitals were able to overcome financial barriers to adding video monitoring technicians to their teams. However, these studies excluded patients with cognitive impairment and assigned them an in‐person observer instead. Arguably, if a remote monitoring technician model was implemented in RAC, a lot of residents would need to be excluded; hence, it is plausible that a return on investment may be unattainable. This might partially explain the lack of adoption in RAC. This inference is not directly supported by data available in the included studies and therefore warrants further exploration in future research. No RCTs, to our knowledge, have directly compared technician‐based monitoring with in‐person observers. Nor have any studies evaluated technician‐based monitoring that provides direct verbal redirection to the patient (where adequate comprehension is required to respond) versus technician‐based monitoring which indirectly raises alerts for a nurse to attend (where patient comprehension is not required).

This review highlights that the U.S. appears uniquely reliant on technician‐based video monitoring—representing 94% of all included studies evaluating this type (Canada representing one other study). Heavily cited reasons why remote video monitoring was attractive in the U.S. were the CMS ruling of non‐reimbursement for in‐hospital falls and the need to reduce costly in‐person observer use. Purvis et al. (2018, 90) claimed “One of the most expensive and controversial safety practices utilised in acute care hospitals in the U.S. is the use of constant observers.” A systematic review on in‐person observers identified that 18 out of the 20 included studies explored alternatives to in‐person observers and all these studies originated in the U.S. (Greeley et al. 2020). The review found that fall rates and injurious fall rates were either lower or unchanged with the introduction of alternative fall prevention interventions (most of which were technician‐based monitoring). Interestingly, the two studies conducted outside of the U.S. introduced in‐person observers, but used volunteers rather than paid staff. Another systematic review, conducted by a team in the United Kingdom, also found research was heavily skewed to the U.S. (Stanton et al. 2025). They examined the decision‐making processes surrounding in‐person observers and ten out of the 16 included studies were U.S.‐based. They identified that the primary reason for streamlining decision making processes was to reduce in‐person observer use. The reliance on in‐person observers in countries outside the U.S. is not clearly described in the literature.

Worldwide, there appears to be growing interest in fall prevention models that reduce the use of in‐person observers. But the rest of the world is not following the U.S. trend towards technician‐based monitoring, for reasons that remain unclear. The U.S. contributed 69% of all global digital health funding in 2024 (Global Overview of Digital Health Funding in 2024 2025), and the co‐location of established video monitoring technology companies in the U.S. may allow better opportunities to develop, test and scale technician‐based systems. Other potential contributing factors may include international differences in funding structures, workforce models, privacy expectations, regulatory requirements or medico‐legal frameworks. Countries outside the U.S. appear to be prioritising smaller evaluation studies of emerging technologies, such as depth‐based automated sensors and portable hand‐held video devices. Thus it may be that these countries are simply at an earlier stage of adopting innovative technological approaches to fall prevention compared to the U.S.

Patient and resident experiences of remote video monitoring appear overlooked in the research base, while staff perspectives dominate. Only 20% of studies were qualitative, and all focus groups were conducted among homogeneous groups. Although mixed stakeholder focus groups can involve ethical challenges, especially related to power dynamics, skilled facilitation can enable patients/residents and staff to share and reflect on each other's experiences or goals with remote video monitoring, generating rich insights to inform the role video monitoring should play in falls prevention.

### 6.1 Limitations

No studies were conducted in low‐income countries, and as highlighted, most were U.S.‐based. This reduces the applicability of findings to hospitals and RAC settings in other cultural contexts with different funding arrangements and medico‐legal drivers. We were able to capture the full breadth of the literature on the use and attitudes towards remote video monitoring for the purpose of falls prevention, however many included studies appeared to lack rigorous methodology. Studies exclusively focusing on effectiveness were excluded from the outset to focus the review on answering the objectives of exploring attitudes and the how and why of remote video monitoring. A systematic review and meta‐analysis of studies investigating remote video monitoring effectiveness would be valuable to inform future recommendations.

## 7 Conclusion

This scoping review highlighted that motivations for remote video monitoring often went beyond pure falls prevention. The effectiveness and acceptance of remote video monitoring are not universal but are deeply context‐dependent, shaped by setting, implementation model, and the perspectives of those involved. There were stark differences between hospitals and residential aged care. In hospitals, remote video monitoring is often considered part of usual care and does not require patient consent, but its use is restricted to those who meet defined eligibility criteria. In contrast, RAC requires explicit consent yet lacks clear inclusion or exclusion criteria guiding who receives video monitoring. Hospitals tend to use systems that involve dedicated technicians or systems that provide automated alerts. In comparison, RAC relies on systems that are only intermittently observed by nurses within their routine duties. Despite the introduction of remote video monitoring as an alternative, hospital nurses appear to have not swayed from their preference for in‐person observers, whilst these are not an option in RAC. Other than technician‐based systems, research is often limited to small pilot studies. Further large‐scale research is required that directly includes patient, resident and family perspectives of remote video monitoring for falls prevention.

## Author Contributions

All authors meet the authorship criteria and approve the content of the manuscript. Conceptualization and methodology: K.S., D.P., J.W.‐N., R.M., and T.P.H. Data collection: D.P., R.M., and K.S. Data curation: K.S. Formal analysis: K.S., D.P., J.W.‐N., and T.P.H. Writing original draft: K.S. Visualisation and Review/editing of manuscript: K.S., D.P., J.W.‐N., R.M., and T.P.H. Supervision: D.P., J.W.‐N., and T.P.H.

## Funding

The authors have nothing to report.

## Conflicts of Interest

The authors declare no conflicts of interest.

## Supporting information


**Table S1A:** Search strategy.
**Table S1B:** Example of Medline search results.

**Table S2:** Eligibility criteria.

**Table S3:** Codes.

**Table S4:** Video monitoring studies characteristics.

**Table S5:** Key quotes depicting themes.

**Data S1:** Preferred reporting items for systematic reviews and meta‐analyses extension for scoping reviews (PRISMA‐ScR) checklist.

## Data Availability

The data that supports the findings of this study are available in the Supporting Information of this article.

## References

[jan70647-bib-0001] Abbe, J. R. , and C. O'Keeffe . 2022. “Continuous Video Monitoring.” Journal of Nursing Care Quality 37, no. 3: 225–230. 10.1097/ncq.0000000000000613. 34983894

[jan70647-bib-0002] Allen, J. A. , R. Reiter‐Palmon , K. J. Jones , L. Sabalka , K. Ciagala , and A. Meens . 2023. “Nurses' Experience Implementing an Automated Video Monitoring System to Decrease the Risk of Patient Falls During a Global Pandemic.” Health 11, no. 18: 2556. 10.3390/healthcare11182556. PMC10530789 37761753

[jan70647-bib-0003] Baez, M. , and C. Karl . 2022. “Preventing Falls With Remote Video Monitoring.” Nursing 52, no. 7: 47–49. 10.1097/01.nurse.0000832384.85335.43. 35752910

[jan70647-bib-0004] Barnett, S. D. , D. Desai , T. Davis , et al. 2020. “Development and Psychometric Testing of the Remote Visual Monitoring Acceptance Tool.” Journal of Nursing Measurement 28, no. 3: 439–454. 10.1891/jnm-d-19-00092. 33199480

[jan70647-bib-0005] Borda, A. , C. Said , C. Gilbert , F. Smolenaers , M. McGrath , and K. Gray . 2020. “Feasibility of Non‐Contact Smart Sensor‐Based Falls Detection in a Residential Aged Care Environment.” In Recent Advances in Intelligent Assistive Technologies: Paradigms and Applications, 165–186. Springer Nature.

[jan70647-bib-0006] Burtson, P. L. , and L. Vento . 2015. “Sitter Reduction Through Mobile Video Monitoring.” Journal of Nursing Administration 45, no. 7/8: 363–369. 10.1097/nna.0000000000000216. 26133097

[jan70647-bib-0007] Centers for Medicare and Medicaid Services . 2008. “CMS Manual System: Pub. 100–07 State Operations Provider Certification (Transmittal 37).” https://www.cms.gov/Regulations‐and‐Guidance/Guidance/Transmittals/downloads/R37SOMA.pdf.

[jan70647-bib-0008] Centre for Reviews and Dissemination . 2025. “How Staff, Patients, Residents, and Families Perceive the Use and Benefits of Mobilisation Alarms in Preventing Falls Within Hospital and Residential Aged Care Settings: A Mixed Methods Systematic Review.” PROSPERO. https://www.crd.york.ac.uk/PROSPERO/view/CRD420251051480.

[jan70647-bib-0009] Cournan, M. , B. Fusco‐Gessick , and L. Wright . 2018. “Improving Patient Safety Through Video Monitoring.” Rehabilitation Nursing 43, no. 2: E6. 10.1097/rnj.0000000000000154. 29499009

[jan70647-bib-0010] Covidence systematic review software . 2025. “Veritas Health Innovation.” https://www.covidence.org.

[jan70647-bib-0011] Davis, F. D. 1989. “Perceived Usefulness, Perceived Ease of Use, and User Acceptance of Information Technology.” MIS Quarterly 13, no. 3: 319–340. 10.2307/249008.

[jan70647-bib-0012] Emilsson, M. , C. Karlsson , and A. Svensson . 2023. “Experiences of Using Surveillance Cameras as a Monitoring Solution at Nursing Homes: The Eldercare Personnel's Perspectives.” BMC Health Services Research 23, no. 1: 144. 10.1186/s12913-023-09130-2. PMC9912642 36765321

[jan70647-bib-0013] Ergai, A. , L. Spiva , S. Thurman , M. Hatfield , M. McCollum , and M. Holmes . 2023. “The Effectiveness of Remote Video Monitoring on Fall Prevention and Nurses' Acceptance.” Journal of Nursing Care Quality 39, no. 1: 24–30. 10.1097/ncq.0000000000000716. 37094580

[jan70647-bib-0014] Fenton, R. , M. Popescu , and D. Moxley . 2016. “Hospital Personnel Attitude Toward Utilization of Depth Imaging Technology in Hospital Rooms.” Missouri Medicine 113, no. 1: 79–84. PMC6139752 27039497

[jan70647-bib-0015] Fischer, A. S. , S. Kaiser , A. Münzing , A. R. Osmaston , M. J. Schmachtel , and M. Sailer . 2024. “Video‐Based Patient Monitoring in Long‐Term Inpatient Care.” Geron 23: 1. 10.4017/gt.2024.23.s.981.opp.

[jan70647-bib-0016] Fitzpatrick, L. 2019. “Is Sensor Technology the Next Step in Fall Prevention?” Nursing Standard 34, no. 12: 56–57. 10.7748/ns.34.12.56.s22.

[jan70647-bib-0017] Geil Kollerup, M. , G. Tolstrup , and B. Schantz Laursen . 2021. “Camera‐Assisted Nursing Observation of Restless Patients in an Acute Care Setting, a Multi‐Method Feasibility Study.” Nursing Open 9, no. 1: 559–568. 10.1002/nop2.1097. PMC8685783 34672427

[jan70647-bib-0018] Global Overview of Digital Health Funding in 2024 . 2025. “Galen Growth.” https://www.galengrowth.com/2024‐digital‐health‐funding‐in‐asia‐pacific‐a‐snapshot/#:~:text=Global%20Overview%20of%20Digital%20Health%20Funding%20in%202024,‐Digital%20Technology%20Innovation&text=The%20United%20States%20led%20the,across%20the%20digital%20health%20landscape.

[jan70647-bib-0019] Greeley, A. M. , E. P. Tanner , S. Mak , M. M. Begashaw , I. M. Miake‐Lye , and P. G. Shekelle . 2020. “Sitters as a Patient Safety Strategy to Reduce Hospital Falls.” Annals of Internal Medicine 172, no. 5: 317–324. 10.7326/m19-2628. 32016286

[jan70647-bib-0020] Hardin, S. R. , J. Dienemann , P. Rudisill , and K. K. Mills . 2013. “Inpatient Fall Prevention.” Journal of Patient Safety 9, no. 1: 29–35. 10.1097/pts.0b013e3182753e4f. 23149692

[jan70647-bib-0021] Hartmann, C. W. , C. Gillespie , G. G. Sayre , and A. L. Snow . 2021. “De‐Implementing and Sustaining an Intervention to Eliminate Nursing Home Resident Bed and Chair Alarms: Interviews on Leadership and Staff Perspectives.” Implementation Science Communications 2, no. 1: 91. 10.1186/s43058-021-00195-w. PMC8383405 34429167

[jan70647-bib-0022] Hawksworth, L. T. , S. Raja , J. Drake , T. Davis , A. O'Brien , and J. M. Brady . 2022. “Exploring Nurses' Perceptions of the Barriers and Facilitators That Influence Acceptance and Use of Remote Visual Monitoring Technology.” Journal of Nursing Management 30, no. 7: 3466–3480. 10.1111/jonm.13822. 36194182

[jan70647-bib-0023] Hebb, A. , M. Kistler , E. George , and B. Zamboni . 2021. “Satisfaction and Technology Acceptance of Staff Regarding Use of Continuous Video Monitoring in Comparison With Sitters.” JONA: The Journal of Nursing Administration 51, no. 2: 60–62. 10.1097/nna.0000000000000970. 33449593

[jan70647-bib-0025] Hu, A. , G. Wood , and J. Hauser . 2024. “Somebody's Watching Me: Video Monitoring of Hospitalized Palliative Care Patients.” Journal of Pain and Symptom Management 67, no. 5: e649–e650. 10.1016/j.jpainsymman.2024.02.095.

[jan70647-bib-0026] Iio, T. , M. Shiomi , K. Kamei , C. Sharma , and N. Hagita . 2016. “Social Acceptance by Senior Citizens and Caregivers of a Fall Detection System Using Range Sensors in a Nursing Home.” Advanced Robotics 30, no. 3: 190–205. 10.1080/01691864.2015.1120241.

[jan70647-bib-0027] Jeffers, S. , P. Searcey , K. Boyle , et al. 2013. “Centralized Video Monitoring for Patient Safety: A Denver Health Lean Journey.” Nursing Economic$ 31, no. 6: 298–306. 24592534

[jan70647-bib-0028] Klymko, K. , L. Etcher , J. Munchiando , and M. Rose . 2016. “Video Monitoring: A Room With a View, or a Window to Challenges in Falls Prevention Research?” Medsurg Nursing 25, no. 5: 329–332.

[jan70647-bib-0029] Latham, K. 2023. “The Impact of Remote Video Monitoring on Costs and Patient Outcomes [Doctoral Dissertation].” https://hsrc.himmelfarb.gwu.edu/son_dnp/129/.

[jan70647-bib-0030] McCurley, J. , and J. Pittman . 2014. “A New Approach to Fall Prevention in Inpatient Care.” Patient Safety & Quality Healthcare 11, no. 6: 50–53.

[jan70647-bib-0031] McNamara, B. 2024. “Nurses' Perceptions of Telemonitoring Devices to Reduce Falls Among Hospitalized Patients: A Literature Review.” Journal of Gerontological Nursing 50, no. 4: 6–10. 10.3928/00989134-20240311-01. 38569107

[jan70647-bib-0032] Morris, M. E. , K. Webster , C. Jones , et al. 2022. “Interventions to Reduce Falls in Hospitals: A Systematic Review and Meta‐Analysis.” Age and Ageing 51, no. 5: afac077. 10.1093/ageing/afac077. PMC9078046 35524748

[jan70647-bib-0033] Nwaise, N. 2017. “Empowering Nurses Through Knowledge and Technology to Decrease Fall Rates [Doctoral Dissertation].” https://scholarworks.waldenu.edu/cgi/viewcontent.cgi?article=5942&context=dissertations.

[jan70647-bib-0034] Peters, M. , C. Godfrey , P. McInerney , Z. Munn , A. Tricco , and H. Khalil . 2020. “Scoping Reviews.” In JBI Manual for Evidence Synthesis. JBI. 10.46658/JBIMES-24-09. 33038124

[jan70647-bib-0035] Popay, J. , H. Roberts , A. Snowden , et al. 2006. Guidance on the Conduct of Narrative Synthesis in Systematic Reviews: A Product From the ESRC Methods Programme. Economics and Social Research Council.

[jan70647-bib-0036] Porock, D. , C. Vafeas , M. Towell‐Barnard , L. Emery , G. Doleman , and J. Alan . 2024. “Cross‐Sectional Survey of Staff and Family Perspectives on the Use of CCTV in Australian Residential Aged Care.” Australasian Journal on Ageing 43, no. 3: 570–580. 10.1111/ajag.13304. 38504424

[jan70647-bib-0037] Pu, D. , K. Stephen , C. McDonald , et al. 2026. “Investigating the Effectiveness of Mobilisation Alarms to Prevent Hospital Falls Using Disinvestment: A Randomised Clinical Trial.” International Journal of Nursing Studies 175: 105320. 10.1016/j.ijnurstu.2025.105320. PMC12825039 41422595

[jan70647-bib-0038] Purvis, S. , A. Kaun , A. McKenna , J. Weber Viste , and E. Fedorov . 2018. “Outcomes of Clinical Nurse Specialist Practice in the Implementation of Video Monitoring at an Academic Medical Center.” Clinical Nurse Specialist 32, no. 2: 90–96. 10.1097/nur.0000000000000356. 29419581

[jan70647-bib-0024] Quigley, B. H. , S. M. Renz , and C. Bradway . 2022. “Fall Prevention and Injury Reduction Utilizing Continuous Video Monitoring: A Quality Improvement Initiative.” Journal of Nursing Care Quality 37, no. 2: 123–129. 10.1097/ncq.0000000000000624. 34231506

[jan70647-bib-0039] Rivera, D. , J. Morelli , N. Barrett‐Fajardo , et al. 2023. “Transforming the Paradigm of Care to Reduce Falls on a Medical‐Surgical Unit Using Artificial Intelligence.” Journal of Informatics Nursing 8, no. 3: 18–28.

[jan70647-bib-0040] Sand‐Jecklin, K. , J. Johnson , A. Tringhese , C. Daniels , and F. White . 2019. “Video Monitoring for Fall Prevention and Patient Safety. Process Evaluation and Improvement.” Journal of Nursing Care Quality 34, no. 2: 145–150. 10.1097/ncq.0000000000000355. 30198947

[jan70647-bib-0041] Sand‐Jecklin, K. , J. R. Johnson , and S. Tylka . 2016. “Protecting Patient Safety. Can Video Monitoring Prevent Falls in High‐Risk Patient Populations?” Journal of Nursing Care Quality 31, no. 2: 131–138. 10.1097/ncq.0000000000000163. 26513398

[jan70647-bib-0042] Stanton, J. , C. Stiles , P. Anderson , R. M. Taylor , C. Vindrola–Padros , and E. Ivany . 2025. “What Decision‐Making Processes Are Used by Nurses in Initiating, Monitoring, and Ending One‐To‐One Observations? A Systematic Review.” Journal of Nursing Care Quality 41, no. 1: 42–48. 10.1097/ncq.0000000000000878. 40341119

[jan70647-bib-0043] Steinberg, K. 2018. “An Alarming Trend.” Caring for the Ages 19, no. 3: 2.

[jan70647-bib-0044] Stephen, K. , D. Pu , J. Weller‐Newton , and T. P. Haines . 2025. “Mobilisation Alarm Use in Hospitals and Alignment With Person‐Centred Practice: A Qualitative Study.” Journal of Advanced Nursing 82: 3489–3508. 10.1111/jan.70113. PMC12994649 40755192

[jan70647-bib-0045] Thomas, J. , and A. Harden . 2008. “Methods for the Thematic Synthesis of Qualitative Research in Systematic Reviews.” BMC Medical Research Methodology 8, no. 1: 45. 10.1186/1471-2288-8-45. PMC2478656 18616818

[jan70647-bib-0046] Tricco, A. C. , E. Lillie , W. Zarin , et al. 2018. “PRISMA Extension for Scoping Reviews (PRISMA‐Scr): Checklist and Explanation.” Annals of Internal Medicine 169, no. 7: 467–473. 10.7326/m18-0850. 30178033

[jan70647-bib-0047] Vandenberg, A. E. , B. van Beijnum , V. G. Overdevest , E. Capezuti , and T. M. Johnson . 2017. “US and Dutch Nurse Experiences With Fall Prevention Technology Within Nursing Home Environment and Workflow: A Qualitative Study.” Geriatric Nursing 38, no. 4: 276–282. 10.1016/j.gerinurse.2016.11.005. PMC6546295 27956058

[jan70647-bib-0048] Vortuba, L. , B. C. Graham , J. C. Wisinski , and A. Syed . 2016. “Video Monitoring to Reduce Falls and Patient Companion Costs for Adult Inpatients.” Nursing Economic$ 34, no. 4: 185–189. 29975024

[jan70647-bib-0049] Woltsche, R. , L. Mullan , K. Wynter , and B. Rasmussen . 2022. “Preventing Patient Falls Overnight Using Video Monitoring: A Clinical Evaluation.” International Journal of Environmental Research and Public Health 19, no. 21: 13735. 10.3390/ijerph192113735. PMC9657748 36360616

[jan70647-bib-0050] Zubrinic, M. , L. Vrbanic , and S. Keshavjee . 2023. “Remote Telemonitoring Is Associated With Improved Patient Safety and Decreased Workload of Nurses.” JTCVS Open 16: 493–497. 10.1016/j.xjon.2023.09.014. PMC10775035 38204626

[jan70647-bib-0051] Zwijsen, S. A. , M. F. Depla , A. R. Niemeijer , A. L. Francke , and C. M. Hertogh . 2011. “The Concept of Restraint in Nursing Home Practice: A Mixed‐Method Study in Nursing Homes for People With Dementia.” International Psychogeriatrics 23, no. 5: 826–834. 10.1017/s1041610210002267. 21208518

[jan70647-bib-0052] Zwijsen, S. A. , M. F. Depla , A. R. Niemeijer , A. L. Francke , and C. M. Hertogh . 2012. “Surveillance Technology: An Alternative to Physical Restraints? A Qualitative Study Among Professionals Working in Nursing Homes for People With Dementia.” International Journal of Nursing Studies 49, no. 2: 212–219. 10.1016/j.ijnurstu.2011.09.002. 21962335

