# Supplementary material for: Attitudes Towards Remote Monitoring for Falls Prevention Among Staff, Patients, Residents and Families in Hospital and Aged Care: Scoping Review
Source: J Adv Nurs. 2026 May 26;82(12):10108–23. doi: 10.1111/jan.70647 (PMC13752629; doi:10.1111/jan.70647)
Supplement: Supplementary file 1 [file JAN-82-10108-s001.docx]

**Supplementary table 1A: Search strategy**

|  | 1 AND 2 AND 3 | | |
| --- | --- | --- | --- |
| **Concept** | **Mobilisation alarms** | **Falls prevention** | **Hospital / residential aged care** |
| Keywords | Mobili?ation alarm* OR Fall alarm* OR Falls alarm OR Fall prevention alarm* OR Falls prevention alarm OR Bed alarm* OR Chair alarm* OR Sensor alarm* OR Bed sensor* OR Bed exit alarm* OR Fall* technolog* OR wearable sensor* OR wearable technology OR equipment alarm system* OR clinical alarm* OR sensor technology OR alarm monitor* OR electronic security system OR electronic surveillance equipment OR assistive technology  OR  tele?monitoring OR (video OR visual OR remote) adj1 (monitor* OR surveillanc* OR observ*) | Fall OR Falls OR Fallen OR falling | Hospital* OR Clinical practice OR Healthcare environment* OR In?patient* OR Subacute OR Acute OR Rehabilitation OR Residential Aged Care Facilit* OR Nursing home* |
| Medline All (OVID) | Clinical alarms/ | Accidental Falls/ | Homes for the Aged/ or Nursing homes/ or Hospitals/ |
| CINAHL complete (EBSCO) | Wearable Sensors  Equipment Alarm Systems | Accidental falls | Residential Care or Long Term Care or Hospitals+ |
| PsychInfo (OVID) | Sensor technology/ | Falls/ | Hospitals/ or Nursing homes/ |
| Embase (OVID) | Alarm monitor/ | Falling/ | Hospital/ or Nursing home/ |
| Emcare (OVID) | Alarm monitor/ | Falling/ | Hospital/ or Nursing home/ |
| AgeLine (EBSCO) | Alarms or electronic surveillance equipment or  or assistive technology | Accidental falls | Hospitals or Hospital care or LONG-term health care (exploded) or  OLD age homes |
| Scopus (Elsevier) | No subject headings | No subject headings | No subject headings |
| Cochrane central register of controlled trials (EBM Review) | Clinical alarms/ | Accidental falls/ | Homes for the Aged/ or  Hospitals/ |

**Supplementary table 1B. Example of Medline search results:**
