# Supplementary material for: Attitudes Towards Remote Monitoring for Falls Prevention Among Staff, Patients, Residents and Families in Hospital and Aged Care: Scoping Review
Source: J Adv Nurs. 2026 May 26;82(12):10108–23. doi: 10.1111/jan.70647 (PMC13752629; doi:10.1111/jan.70647)
Supplement: Supplementary file 3 [file JAN-82-10108-s003.docx]

**Supplementary table 5. Key quotes depicting themes**

| **Theme** | **Quote** |
| --- | --- |
| **Effectiveness** | ‘We have a lot of patients on our unit that are confused … or come in with withdrawals, and it’s really good … [by] either giving them [the] cues that they need like please call your nurse or stay in the bed.’ Hospital (Hawksworth et al., 2022)  ‘You just have patients that … you have to be there, one-on-one for them to be able to follow the command. … you put an [RVM] [but] they do not even follow whatever you say.’ Hospital (Hawksworth et al., 2022)  ‘Initially I was very skeptical and not at all a fan, but they (the cameras) have proved much more helpful than I anticipated. When (RVM) CNAs speak to the patients, we hear it and can go in before anything actually happens. Much like a bed alarm, it gives us an extra 60 second warning before a fall.’ Hospital (Jeffers et al., 2013)  ‘There’s a difficulty about the timeliness of the staff. Yes, we’re aware the bell goes off, yes we’re aware people are on the move, but it’s what’s also happening in the building at that particular moment in time.’ RAC (Borda et al., 2020) |
| **Workload impact** | “For staff it’s an experience, which will help to manage our time on the ward and help to prioritize our work” Hospital (Wright & Singh, 2022 linked to Fitzpatrick, 2019 study)  ‘You can prioritize in a completely different way when you have cameras, because then you can see. If there are several bed alarms at the same time, for example, then you can see, ‘no, that was just the blanket, but there, Greta is up’. Then you can run to her, rather than to run and wake up the person where it was just the blanket and get him up too.’ RAC (Emilsson et al., 2023) |
| **Liability concerns** | ‘One [colleague] thought that the cameras were on all the time. Well, shall we be there then? Some felt uncomfortable that we should maybe be on film, did someone sit and watch us?’ RAC (Emilsson et al., 2023)  ‘I feel I am to blame if the patient falls when I am not present.’ (Geil Kollerop et al., 2021) |
| **Device knowledge** | ‘No, no education. We just went through how it worked at the introduction, so no training other than that.’ RAC (Emilsson et al., 2023)  ‘I think what creates the bias against the machine is when it does not work and everybody tends to say all the negative things that have happened when we have used it … instead of all the good that it’s done, or [to] help keep the nurses where they need to be- at the bedside.’ Hospital (Hawksworth et al., 2022) |
| **Ethical tensions** | And the other thing is… the angle of view…at times, it’s in the middle of the room with our two roommates…So, maybe also looking at the other[patient], which they might not even need or want…’ Hospital (Hawksworth et al., 2022)  ‘It’s important for the relatives to know your mother is not cared for by a robot.’ Hospital (Geil Kollerup et al., 2021)  ‘Reduces the homelike environment and promotes a custodial/surveillance environment.’ RAC (Porock et al., 2024)  ‘The range images do not make me feel as though I’m being watched.’ RAC (Ilo et al., 2016)  Falling was ‘less important than taking people’s freedom away.’ RAC (Vandenburg et al., 2017) |
