# Supplementary material for: Attitudes Towards Remote Monitoring for Falls Prevention Among Staff, Patients, Residents and Families in Hospital and Aged Care: Scoping Review
Source: J Adv Nurs. 2026 May 26;82(12):10108–23. doi: 10.1111/jan.70647 (PMC13752629; doi:10.1111/jan.70647)
Supplement: Supplementary file 4 [file JAN-82-10108-s004.docx]

**Supplementary Table 3: Codes**

| **Video monitoring type** | **Motivation to implement video monitoring** | **Effectiveness** | **Workload impact** | **Applied device knowledge** | **Liability concerns** | **Ethical tensions** |
| --- | --- | --- | --- | --- | --- | --- |
| Remote technician-based video monitoring systems  Automated video monitoring systems that generate alerts  Nurse-observed video monitoring systems without alerts | Falls prevention  Workforce optimisation  Cost effectiveness  Enhancing care experience | Sense of security  Ability to respond in time  Patient type  Inevitable falls  “Extra eyes”  No substitute for staff  Unmet needs  Imperfect technology | Decision making algorithms  Communication between VMT and staff  VMT alert threshold / alarm fatigue  Set-up & monitoring  Explaining system to user  Saves unnecessary checks/investigations  Job satisfaction  Teamwork | Technical knowledge – VMT and nurse  Clinical knowledge  Evidence based knowledge  Policies & procedures  Ease of use  Patient & family education  Changing culture | Expectations to use  Policies  Professional reputation  Data abuse  Watched and judged | Risk-benefit analysis  Justice  Autonomy  Consent  Privacy  Data abuse  Functional restraint |
