# Supplementary material for: Attitudes Towards Remote Monitoring for Falls Prevention Among Staff, Patients, Residents and Families in Hospital and Aged Care: Scoping Review
Source: J Adv Nurs. 2026 May 26;82(12):10108–23. doi: 10.1111/jan.70647 (PMC13752629; doi:10.1111/jan.70647)
Supplement: Supplementary file 5 [file JAN-82-10108-s005.docx]

**Supplementary table 4: Video monitoring studies characteristics**

| **Author** | **Study aim** | **Key findings** |
| --- | --- | --- |
| Abbe et al., 2022 | To identify data metrics to inform expansion of VM programs when determining readiness for growth | - > 94% staff satisfied or neutral with VM capabilities related to patient safety, decision to invest in VM and believed additional VM investment necessary  - Within 1^st^ year of VM, utilization remained > 80% for all 10 mobile units and 3 stationary ceiling mounts. 10 mobile units in use 90 - 95% of the time  - Wait-list data showed 2 to 3 patients waiting for available cameras 33% of the time, 3+ patients waiting 33% of the time, and 33% of the time there was no wait-list. 164 instances of patients on the wait-list who had a sitter but were unable to fulfill the VM request |
| Allen et al.,  2023 | To evaluate nurses’ attitudes toward AVMS implementation over time in two units of an acute care hospital after a customized training program | - Perceptions of usefulness of AVMS improved over time during intervention  - Perceptions of social influence support AVMS improved over time  - At time two, only social influence related to usefulness. At time three, only behavioral control related to usefulness  - At the height of COVID, many nurses indicated implementing VM was not a priority |
| Baez & Karl,  2022 | To identify nursing staff perceptions on the use of RVM, collect fall data following the implementation of RVM, and determine the benefits of using RVM in reducing fall rates | - 85% perceived RVM a valuable adjunct intervention for patient safety  - 77% perceived RVM an effective fall prevention method  - 81% believed RVM assisted with fall prevention - only 68% said RVM prevented between 1-3 falls while their patients were under surveillance  - 81% experienced a sense of security  - 83% expressed increased satisfaction with RVM |
| Barnett et al., 2020 | To describe the development and psychometric testing of the RVMAT (Remote Video Monitoring Acceptance Tool) | - Overall high level of RVM endorsement (3.73)  - Item means ranged from 1.83 to 4.05  Lowest: "I believe a sitter is safer than RVM"  Highest: "Having RVM available for patient monitoring is valuable" |
| Borda et al.,  2020 | To investigate the feasibility of the implementation of a NCSSS to monitor behaviour and detect falls in a RAC | - Sensors did not alter staff’s relationship or frequency of contact with residents because they were already needing more assistance and regular checking  - Despite start-up issues, it is worth persisting with using technology for monitoring behavior and falls detection  - Facilitates staff to be more proactive to prevent falls. Excited where research is leading  - Residents not observed concerned about visual images. Stick figures good for privacy and confidentiality |
| Burtson & Vento, 2015 | To significantly decrease annual sitter staffing cost below the level of 2012 expense while maintaining or improving patient safety | 1) VM guidelines (patient criteria)  2) VMT training (competency checklist)  3) House-wide education  4) Operational plan for standardized workflow - referral procedure, documentation, escalation, VMT breaks  - Hesitation to use VM for patients currently with sitters. A sitter remained for 1st few hours of VM and intervened only if VM redirection was unsuccessful. |
| Cournan et al., 2018 | To compare rates of falls before and after implementation of RVM, in addition to quantifying costs saved as a reduction in falls and one-to-one sitter usage | - 81% staff indicated either a general or strong favorable reaction to RVM  - 91% felt RVM was not intrusive. 4% indicated monitors were a privacy intrusion  - 5% answered the monitors were physically intrusive, took up too much space. Portable cameras are bulky and take up valuable space but can be easily moved between rooms. Fixed cameras/speakers take up less space, but do not allow for flexibility when placing patients. Multiple room changes may be required - time-consuming and impacts patient satisfaction |
| Emilsson et al., 2023 | To analyze obstacles and opportunities associated with implementation and use of surveillance cameras at nursing homes from perspectives of staff | **Prerequisites for implementation:** Need for adequate education; Importance of functionality; Need for support  **Improvements in night working environment:** Easier for staff to help each other; Easier to prioritize  **Balance between security and integrity:** Ability to not disturb unnecessarily; Not a substitute for human presence; Importance of integrity |
| Ergai et al.,  2024 | To examine RVM effectiveness as a strategy to decrease patient falls and investigate nurses’ acceptance and perceived usefulness of RVM technology | - Moderate satisfaction with RVM (3.38)  - RVM and Patient Safety: 3.26; Attitude toward using RVM: 3.33; Perceived Usefulness of RVM: 3.41; Intention to Use RVM: 3.43; Perception of Ease of Use of RVM: 3.57  - Lead VMT responsible for assigning patients to VMTs, monitoring acuity of assignments, and lunch/break relief  - VMT facility liaisons provide support to the VMT team by providing in-person interaction with monitored patients and setting up/removal of camera. Facility liaisons use a sitter order panel in the EHR to identify patients they are supporting and documenting when handing them off to the next shift |
| Fenton et al., 2016 | To determine hospital personnel perceptions toward utilization of depth imaging technology in hospital rooms and to explore expanded use of video surveillance systems in the patient room | **Group A: Leadership:** More optimistic than apprehensive. Focused on financial impact, liability, risk-to-reward ratio, educational opportunities, process improvement, training capability, auditing and monitoring capability, compliance improvement among staff and patients, and as a mechanism to improve work injury programs. Concerns raised regarding privacy, initial investment cost, and staff buy-in  **Group B: Physicians:** More hesitant. Adopting a moderate position. 83.3% felt it would be intrusive to employee and patient. Physician privacy concerns were higher than other groups  **Group C: Nursing:** Overwhelmingly positive. Variances in opinion associated with privacy concerns  **Group D: Therapists:** Positive. Moderate hesitancy regarding privacy. Could benefit care planning, but concerned about productivity and privacy invasion  **Group Results:** All groups rated potential moderate positive impact. Average rating 3.4/5 – moderate**.** No direct experience with VM, but were open to idea of implementation. Depth image was an acceptable format to maintain privacy. Potential negative impacts were issues of liability and ease of use |
| Fischer et al., 2024 | To conduct qualitative and quantitative data as a basis for  discussion and critical analysis for institutions preparing implementation process of VM systems | - 47.1% (n=115) survey participants in favor of VM; 40.1% (n=98) undecided  - 75.8% (n = 185) believe VM should not be used across the board and specific groups of people should be considered differentially  - Concerns: data protection, restriction of privacy and self-determination, fear of data abuse  - Advantages: vital sign monitoring, faster reaction in emergency, reduction in nurse workload, increase patient safety through improved fall prevention  - Authors concluded a general interest in VM implementation is apparent. If there is a lack of acceptance, the assumption could be made there are too few supporters and VM would not find its way into everyday nursing care. Further research required including perspectives of those receiving VM |
| Fitzpatrick 2019. Linked paper:  Wright & Singh, 2022 | To report interim findings of using a sensor to detect patients' movements | Staff perceptions of the system became increasingly positive:  - July 2018: 79% agreed or strongly agreed that ‘the system has enabled me to prevent a fall’  - October 2018: 92% agreed or strongly agreed that ‘the system has enabled me to prevent a fall’  - Staff feel the technology makes patients safer and provides greater job fulfilment. Better care decisions because able to view what would previously have been unwitnessed falls. Carers report greater peace of mind when the technology is used |
| Geil Kollerup et al., 2021 | To evaluate the implementation, practicality and acceptability of camera- assisted nursing observation for restless patients at the acute care ward | **1. A potential tool for improved patient safety** – prevent falls, adjust care to match patient needs  **2. Affecting nurses’ working conditions** – provides peace of mind, increasing confidence and overcoming stress when working with restless patients, stress could increase if the nurse was unable to act on a critical situation observed on their phone while caring for another patient  **3. Requiring ethical considerations** – Caring and watching the phone at the same time might hamper the nurse's ability to give a patient their full and undivided attention - a prerequisite in establishing a trusting relationship and helping the patient to feel safe  - The camera was not directed at the face to maintain privacy but ability to see the face would have given extra cues of the patient’s emotions or their attempts to communicate to assist nurses deciding whether they needed to intervene  - Do not need to repeatedly check and disturb sleeping - able to monitor patients with delirium from outside the room reduces noise and disturbance.  - Nurses reported families appeared positive towards VM – family perspective not directly measured  - 94% agreed that camera-assisted observation should be a possibility in the acute care ward |
| Hardin et al., 2013 | To compare patient falls on medical-surgical units with and without Webcams, and assess use of the Morse Risk Assessment (MRA) for identification of high risk for injuries | **Patient thought to benefit from Webcam virtual bed rails:** Confusion, dementia, delirium, postsurgical within the first 24 hours, psychiatric awaiting medical clearance, TIA/stroke, alcohol withdrawal or impaired mobility  **Patients physicians thought benefited:** Confused, dementia, altered mental status, electrolyte imbalance, or alcohol withdrawal. Some physicians transferred patients to the study unit to access virtual bed rails.  **Advantages and disadvantages of Webcams:**  - Improved nursing confidence to provide rapid response, especially for those not near nursing station. A valuable substitute for sitters  - Negative effect: diminished patient and physician privacy. Nurses concerned Webcams would be used to monitor their work  - Acceptance less problematic if it was policy all patients with 50+ risk score required Webcams/virtual rails, and patients informed of this on admission. Less refusal rate when Webcams presented as ‘‘usual care’’ in other facilities and patients had to actively refuse  - Structured staff and patient education required.  - The system needed refinement. Reliability was an issue, and promptness of responses by Customer Service Department was a concern. Time-consuming to add or delete a patient from having camera on. It would be economical to have designated Webcam rooms with virtual bed rails for high-risk admissions |
| Hawksworth et al., 2022 | To better understand direct care nurses’ and nurse leaders’ perceptions of barriers and facilitators that influence nurses’ acceptance and use of RVM technology | **Contextual human factors impact nurse acceptance**  - Patient factors: RVM helpful for re-directable patients. Not helpful for quick, agitated, hallucinating patients who cannot follow commands  - RVM technician (RVMT) factors: Monitoring style / skill, communication to team, comfort with escalating, partnering up, communication to patients  **Facilitators and barriers related to RVM’s functionality**  - All groups agreed RVM had capacity to be useful. It provided an “extra set of eyes”  - Language: Tone, Pre-recorded, Volume, Voice preference, family’s voice, Language preference  - Visual capabilities: Dim light, View angle, Privacy in semi-private rooms with patients not using RVM  - Connectivity: Losing connection, work flow impact because unaware when system will come back  **Nurse leaders’ role in maintaining device availability and efficient use**  - Lack of availability, inefficient use, simultaneous use of sitters  **Nurse leaders’ role in promoting adoption of the technology**  - Clarifying RVM protocol: To guide proper use of sitters v RVM  - Educating, system familiarity, sharing expectations, staff educate patients and family  - Addressing nurse preference for sitters: Culture, pushbacks, person v machine, reassessing need for sitters, explaining trickle effect, advocate, encourage  **Leaders’ role in valuing nursing professional judgement**  - Balancing guideline adherence with valuing nurses’ assessment of client situation  - Leadership influence/style: Ignoring nurses’ judgement, ensure nurses feel heard, shared decision-making  - Concern RVM needed to be trialed according to protocol and patient must ‘fail’ thus risking harm |
| Hebb et al.,  2021 | To determine staff satisfaction and technology acceptance of RVM in comparison to sitters | - RVM is used on a patient on my unit every shift: Yes: 14 (25.93%); No: 32 (59.26%); Sometimes: 8 (14.81)  - RVM is just as effective as a safety sitter: Yes: 7 (12.73%); No: 27 (49.09%); Sometimes: 20 (36.36%); Don't know: 1 (1.82%)  - Satisfied with communication from RVM through command center calling me or STAT alarm that a patient needs assistance: Yes: 26 (47.27%); No: 22 (40%); Sometimes: 6 (10.91%); Don't know: 1 (1.82%)  - I feel satisfied with education received on RVM when it was 1st initiated: Yes: 29 (53.7%); No: 7 (12.96%); Sometimes: 16 (29.63%); Don't know: 2 (3.7%) |
| Hu, 2024 | To describe potential benefits and harms of VM of hospitalised palliative care patients. | - Expressed mistrust of room camera, stating VM impacted emotional well-being, sleep, and led to a perception all medication intake was monitored  - Patient had a makeshift covering placed on the camera during a previous admission. Camera disabled but not permitted to be covered per hospital policy in current admission. Despite assurances, patient refused to leave unmonitored bathroom, refused nursing assessments, and threatened to leave against medical advice. VM fractured previously trusting relationships with palliative care team |
| Iio et al.,  2016 | To investigate user evaluations about senior and caregiver intentions to use and feelings of security of a fall detection system in nursing homes  Hypothesis:  A fall detection system using range sensors is accepted by seniors more than commonly used fall detection systems | **First phase:**  **Staff issues with current sensor:** False alerts - alarm fatigue and work disruption; Some seniors avoid the sensors; Malfunctioning alarms  **Functions needed in fall detections?** Senior’s acceptance - comfort, security and privacy; Appearance view - to know senior’s status as soon as possible to prioritise action; Fall detection area - to detect anywhere not just near bed  **Second phase:**  **Intention to use - older person:** 4.30 out of bed sensor; 5.86 fall detection with constant observation**;** 6.04 fall detection without constant observation  **Feelings of security - older person:** 4.69 out of bed sensor; 6.38 fall detection with constant observation; 6.23 fall detection without constant observation  **Intention to use – staff:** 3.66 out of bed sensor**;** 5.78 fall detection with constant observation**;** 5.56 fall detection without constant observation  **Feelings of security – staff:** 4.71 out of bed sensor; 5.85 fall detection with constant observation; 6 fall detection without constant observation  **Older person Sensor preference:**  Out-of-bed sensor not enough, unsafe or unnecessary 12.7%  Out-of-bed sensor is enough and safe 12.7%  Constant monitoring is best 26.4% - A majority would like to be monitored all the time valuing security more important than privacy  Monitoring only for danger situations is best 18.2%  Switching monitoring level depending on situation: 5.5% - keen for easiness to change fall detection functions according to health statuses of seniors  Privacy-minded, but acceptable because of safety 1.8% |
| Jeffers et al.,  2013 | To describe VM as an innovative approach to address organizational demand to ensure patient safety at reduced operational expenses | - RVM program met with skepticism initially by NMs and staff. However, shortly after implementation, value of RVM became apparent and is now widely accepted. "Initially I was very skeptical and not at all a fan, but they (the cameras) have proved much more helpful than I anticipated. When (RVM) CNAs speak to the patients, we hear it and can go in before anything actually happens. Much like a bed alarm, it gives us an extra 60 second warning before a fall," stated nurse manager. A VMT noted, "This has been a great way to enhance patient safety while maintaining privacy." |
| Klymko et al., 2016 | To explore antecedents to falls in acute setting from lived experiences of a diverse sample of expert health professionals, patient care assistants (1:1 safety sitters), VMTs, nurses and fall prevention experts | **Structural issues within the room:** It's confusing for RVM patients to hear the VM voice (audio)  **Process issues within the room:** VMT monitors more closely for 5 mins after staff leave room because patients may reconsider their needs and try to walk  **Behaviour exhibited by patient:** The patient may "dead stare into the camera" as if to say "Okay, who's going to see me here, can I get away?" Agitated or confused patients often needed fears allayed  **Antecedents appearance during RVM:** "It's not as clear ... you can't really see what they're doing exactly." However, RVM provided a view otherwise unavailable unless in the room. RVM distortions: room appearing "far away", partial visualisation of room, patient movements appear "slow motion", lack of clarity despite infra-red capability in the dark and patient behaviours appearing accentuated. These distortions led staff to respond to observed patient movement by using audio communication to the room or sending staff to the room  - Audio communications could precipitated behaviours leading to a fall e.g. sleeping patient awakened with confusion and fear of "intercom voice"  **Environmental conditions and patient behaviours prompting action:** Changes in: environment - lighting, sound, temperature; environment - loose cords, wet floors, objects out of reach, room changes; process based factors - shift changes, meal times, awakened from sleep; patient based factors - soiled, pain, anxiety |
| Latham, 2023  THESIS | To evaluate the effectiveness of RVM compared to standard care (1:1 observation) on: falls, sitter use and costs, patient and staff satisfaction | - No difference in patient satisfaction scores between pre / post periods - both units trended a decrease in their scores.  - Post RVM, staff had statistically significant higher mean survey ratings on questions regarding patient safety and monitoring with RVM (t(175)=-2.733, p=.003), effectiveness of RVM compared with a live sitter (t(175)=-1.824, p=.035), and ease of use of RVM (t(175)=-3.434, p<.001) |
| McCurley & Pittman, 2014 | Not explicitly stated  To find a solution to issues associated with increased sitter usage | - Feedback was uniformly positive. Nurses and PCTs often see sitting as a distraction from patient care, while staffing and managing sitters is an ongoing challenge. Chief medical officer championed RVM and served as a liaison to medical staff, answering questions related to RVM  - VMT initially doubted RVM ability to prevent falls compared to in-person sitter, but now views RVM as effective  - RN viewed instant ability to commence RVM advantageous compared to delays sourcing in-person sitters. VM preferable as VMT can talk to the patient and request them to wait for nurse and alert nurse early if patient at risk rather than bed alarm which cannot talk to patient and alerts only on patient movement  - Family member supported RVM use and experienced prompt responses from nurses. Reassured when they were not visiting that there was monitoring |
| Nwaise, 2017  THESIS | To increase nurses’ knowledge on proper use of CVVM as an attempt to effectively decrease fall rates | **Knowledge test:** - Knowledge of criteria for monitoring a patient using RVM: Pre: 34.4%; Post: 90.6%  - Knowledge on hospital policy on RVM of qualifying patients using RVM: Pre: 3.1%; Post 65.6%  - Card (containing info on RVM) placed on patient tables contains useful information: Pre: 13.2%; Post: 71.1%  **Attitudes:** - More knowledgeable on RVM use to prevent fall: 93.3% Strongly agree / agree, 6.7% missing  - More likely to always use RVM to prevent fall: 93.3% Strongly agree / agree, 10% disagree, 6.7% missing  - More confident using RVM: 93.3% Strongly agree / agree, 6.7% missing  - Believe RVM in addition to other fall prevention strategies prevent falls: 93.3% Strongly agree / agree, 6.7% missing |
| Porock et al.,  2024 | To describe staff and family members' opinions about CCTV in communal and private areas of RACFs, and to investigate how this relates to perceptions of care quality | - Most staff and families felt there were advantages to using CCTV in public areas for resident and staff safety, fall monitoring and prevent abuse. Staff and family members reported advantages of CCTV in private areas with similar reasoning  - Staff identified disadvantages to CCTV monitoring in public areas. Fewer family members had the same concerns.  - Most staff and family recognised disadvantages of using CCTV in private areas  - Staff and family felt ‘facilities are residents’ homes' and privacy may be threatened by CCTV in public and private areas. 11 (14%) staff said they may feel ‘watched or judged’ by monitoring in public areas  - Family expectations were that CCTV was continually monitored by staff or external security and therefore, real-time staff responses to incidents possible |
| Purvis et al.,  2018 | To design and implement a Constant Observation (CO) program that utilizes video cameras with the goal of monitoring for and preventing unsafe patient events that can lead to poor outcomes | - Patient and family involvement key to success. A patient education document of the CO Program presented to the Patient and Family Advisory Council who were positive. Advised to outline to families that VMTs would be trained CNAs and no recording is done - integral to patient/family acceptance  - Concerns arose during implementation: alarm volume, when the alarm should be activated, and when verbal redirections should occur. These concerns were addressed by encouraging communication between the inpatient staff and the VMT in established change-of-shift reports  - Important to address privacy concerns of other disciplines via providing information from other facilities using RVM and providers seeing VMTs at work  - Safe transition to RVM was a concern. Nurses often felt patients with PSAs required too many redirections for the VMT to maintain safety. A RVM trial option was implemented where the RVM was used in conjunction with a PSA for 3 to 4 hrs. |
| Quigley et al., 2021 | To implement virtual sitters utilising VM in hospitalised patients | - Processes developed: Education; Policies; VMT Job descriptions; Algorithm to guide judgement, inclusion/exclusion criteria  - Nursing leadership saw the value in RVM and thereby increased the usability in this facility  - Favorable feedback from staff, patients, families, nurse champions, and management received |
| Rivera et al., 2023 | To assess the influence of Artificial Intelligence Sensor on fall and fall with injury rate, Bed shift report, and hourly rounding on a medical surgical unit | No statistical difference between pre and post ratings on any of the nurse survey items administered as part of pilot trial evaluating a new AI based sensor:  - Ability to provide a safe environment of care with the tools and technology available (bed alarms, Posey equipment, BMAT, fall interventions).  - Work effectively and efficiently without multiple interruptions related to patient safety.  - Work collaboratively with other members of the team to provide a patient care environment that promotes safety.  - Able to purposefully round on my patient in a timely manner to ensure that they have everything they need for a safe care environment.  - Satisfied with the tools that we use to provide safe care for our patients who are at risk for fall. |
| Sand-Jecklin et al., 2019 | - Identify nursing staff and VMT perspectives on VM process  - Identify patient/family perceptions related to VM  - Revise and standardize process for VM and optimize its value for protecting patient safety | - RVM perceived by all groups as helpful in protecting patient safety and preventing falls  - RVM process itself applied inconsistently or needed to be delineated more clearly  - Overhead paging of staff alerts for high risk situations perceived as most effective VMT intervention; however, VMTs indicated this option not always available, as only 1 overhead page could be called at a time due to technology limitations  - VMTs hesitant to verbally redirect patients due to it seemed to startle or frighten them  - Need for increased communication and additional information about patients identified frequently by VMTs but to a lesser degree by nursing  - Patient/family did not perceive RVM as troublesome but indicated increased communication about monitoring process was needed |
| Sand-Jecklin et al., 2016 | To describe the process of implementing centralized video monitoring (CVM) as a fall prevention strategy and to document the outcomes as they relate to patient falls, VMT behaviors and use of sitters | - 1508 cases of RVM on all units during the study, 697 for first 2 units and 811 for second units. Average number of monitored days per patient: 3.3 (median1.0), with a positive skew, indicating a very small number of patients were monitored for many days, while most were monitored for only 1 day  - VMTs called staff: average of 7 times per patient (range: 0-346). Overhead pages: less common, medians of 0 for both data collection periods and means of 1.5 and 2.4 (range: 1-320). VMT redirection of patient via camera microphone: less often, mean 1.2 and 0.5 (range: 0-124). There was a positive skew to all data, indicating for a small number of patients, there were a large number of staff calls, overhead pages, and redirections.  - 5% of video-monitored patients also required sitter  - The process was well accepted by nursing and other hospital personnel, as well as by patients and families |
| Vandenberg et al., 2017 | To examine how existing fall prevention technology was experienced within nursing home nurses’ environment and workflow | **U.S: “Everybody’s supposed to be listening” Falls a problem (used traditional mobilization alarms)**  - Staff rely on their senses and cannot coordinate care. Staff may hesitate, unaware where alarm is alerting from. Chaos provoked among staff as they answer alarms and leave other residents unattended. Staff cannot tell who was taking care of the problem until arriving on scene  - Alarms faulty, broke, or easy to dismantle by residents, alerted too late to prevent falls, and emitted a piercing noise that inhibited movement, caused agitation and haunted staff  - More staffing wanted. Suspicious of potential technological solutions such as cameras fearing they could be used to blame staff for wrong-doing  **NL: “I just feel normal, during night shift too” Rarely encountered falls (used combination of alarms including video monitoring)**  - Relaxed attitude towards falling from management. Problem of falling “less important than taking people’s freedom away.”  - Safety culture of individualized care with delegated and coordinated responsibilities enabled by technology and care unit configuration. Make agreements with residents about tradeoff between safety and freedom. Agreements recorded in care plans.  - Nurses in psychogeriatric units have cameras in common areas of 10 small homes at night |
| Vortuba et al., 2016 | To evaluate the effectiveness of RVM with a dedicated telesitter in order to reduce falls, as well as to reduce patient companion usage | - Average length of time for each RVM event: 2.5 days. Length of time on RVM ranged as high as 28 days  - VMT redirected patients via the microphone 5,413 times (average 10 times/shift). VMT made 5,880 calls to caregivers (average of 11/shift)  - An area for further study is accurate criteria for selecting the most appropriate patients for VM and the most appropriate length of shift for a VMT and the most effective VMT-to-patient ratio |
| Woltshe et al., 2022 | - To investigate nurses’ perceptions of PVM (Passive Video Monitor)  - To evaluate effectiveness of overnight PVM as a falls prevention strategy for high fall risk patients in inpatient clinical units | - No patients declined PVM  **PVM taken on nursing rounds:**  - Ward A: Yes: 49 (34.8%) No: 92 (65.2%)  - Ward B: Yes: 170 (85.4%) No: 29 (14.6%)  - Ward C: Yes: 143 (92.9%) No: 11 (7.1%)  - Yes: 362 (73.3%) No: 132 (26.7%)  **Staff satisfaction:**  - Like using PVM overnight: Ward A: 92% Ward B: 100% Ward C: 100% Total: 97%  - Been alerted to a patient as a result of PVM: Ward A: 57% Ward B: 100% Ward C: 100% Total: 80%  - Believe PVM prevented falls overnight: Ward A: 71% Ward B: 100% Ward C: 100% Total: 87%  - Feedback predominantly positive, PVM a helpful, valuable tool, easy to implement and use  - Monitor screens too small, difficulty identifying patients or room numbers on small screen, limited battery life and connectivity issues  - Implementation of a larger screen, whilst helpful to visibility, would reduce portability of PVM |
| Zubrinic et al., 2023 | To evaluate the impact of RVM technology in our hospital network through a quality improvement lens as it relates to patient safety outcomes and nursing workload | - RVM provided 286,538 hours of remote patient observation, averaging 148.5 hours (6.25 days) per patient  - Median duration: 61.5 hours (2.5 days) per patient. One patient monitored for extended period of 9325.75 hours (388.5 days)  - Various services used RVM to monitor patients: 42% (n = 825) medicine, 37% (n = 711) surgery, and 21% (n = 417) rehabilitation/complex care  - VMTs completed a total of 54,716 interventions, including 8427 managed using audio redirection only without requiring nurse to attend bedside  - RVM considered on a case-by-case basis for patients who do not speak English, are deaf or severe delirium, dementia or confusion and not re-directable  - Patients are not placed on RVM if they are already monitored by a sitter, or have auditory hallucinations  **- Nursing** feedback predominantly positive. More comfortable knowing someone was monitoring patients they were concerned about, RVM helped managing multiple impulsive patients and patients in rooms not in high-traffic areas. Some nurses experienced difficulties answering calls from VMTs while busy  **- Family** felt more at ease leaving the hospital, knowing a VMT was monitoring and addressing their family member’s needs  - **Patients** reported feeling scared, isolated, and vulnerable in hospital, but having a VMT provided reassurance someone was watching over them and could call for help quickly if necessary. Patients reported RVM was not intrusive and felt no different from any other equipment in the room to keep them safe |
| Zwijsen et al., 2012 | To examine the views of professionals in dementia care on surveillance technology as an alternative to physical restraints. How is surveillance technology currently being used in Dutch nursing homes? What are, according to the professionals, the pros and cons of using surveillance technology in nursing home care? | **Providing additional safety:** - Provides a feeling of extra safety. Tempting to use surveillance on almost all residents. Staff did not regard surveillance technology as harmful. Felt they should be able to place devices whenever they felt it could give them or others more piece of mind about resident safety  **Providing more freedom for residents:** Prevents need to disturb residents’ sleep or daily activities. Nurses can rely on the devices to tell them when to act  **Limitations of surveillance technology:** It helps when staff know residents well to prioritise response  - Surveillance could violate privacy: Some who worked in homes that used a lot of surveillance expressed privacy concerns but this had not led to action to reduce use  - Participants did not raise concerns previously raised in research e.g. impact quality of care relationship by reducing moments of face to face contact - cared 'from a distance', loss of dignity through stigmatisation |
| Zwijsen et al., 2011 | To explore how care professionals and family members of RAC residents with dementia in the Netherlands experience and define the concept of restraint | - Over half of respondents did not regard surveillance technology devices as restraints  **Criteria used to define an intervention as a restraint:**  The perception of the resident: If a resident does not notice an intervention, it cannot be regarded as a restraint; A resident may notice the intervention, but does not experience any inconvenience because of it; Advancing dementia complicated interpretation of resident - problematic when resident’s perception is used to define restraining qualities of interventions  The intention of the care professional: The most frequently mentioned reason for using an intervention concerns the safety (i.e. falls) of the resident. The intention of interventions is not to restrict the resident, but to protect them from harm or give them help they need and therefore not labeled as restraints. Invasion of privacy: In defining surveillance technology as restraint, an extra criteria was used that was not applied to other types of restraints - privacy concerns. Relatives did not regard monitoring devices as restraint. Were surprised others did  Differences between respondents: Nurses and relatives felt in some situations there was no choice but to use a restraint. However, key persons, who were mostly nursing home physicians, did not always feel restraints should be used  - Authors advised it was important to be aware of the “local logic” of care practice and take into account that staff and relatives regard an intervention as a restraint only when it is bothering a resident or when it is used for the sole purpose of restricting freedom and/or when it invades resident’s privacy |
